# Modes of Selection in Tumors as Reflected by Two Mathematical Models and Site Frequency Spectra

**DOI:** 10.3389/fevo.2022.889438

**Published:** 2022-08-01

**Authors:** Monika K. Kurpas, Marek Kimmel

**Affiliations:** 1 Department of Systems Biology and Engineering, Silesian University of Technology, Gliwice, Poland; 2 Department of Statistics and Bioengineering, Rice University, Houston, TX, United States

**Keywords:** Moran model, cancer evolution, drivers and passengers, drift and selection, simulation, breast cancer, DNA sequencing

## Abstract

The tug-of-war model was developed in a series of papers of McFarland and co-authors to account for existence of mutually counteracting rare advantageous driver mutations and more frequent slightly deleterious passenger mutations in cancer. In its original version, it was a state-dependent branching process. Because of its formulation, the tug-of-war model is of importance for tackling the problem as to whether evolution of cancerous tumors is “Darwinian” or “non-Darwinian.” We define two Time-Continuous Markov Chain versions of the model, including identical mutation processes but adopting different drift and selection components. In Model A, drift and selection process preserves expected fitness whereas in Model B it leads to non-decreasing expected fitness. We investigate these properties using mathematical analysis and extensive simulations, which detect the effect of the so-called drift barrier in Model B but not in Model A. These effects are reflected in different structure of clone genealogies in the two models. Our work is related to the past theoretical work in the field of evolutionary genetics, concerning the interplay among mutation, drift and selection, in absence of recombination (asexual reproduction), where epistasis plays a major role. Finally, we use the statistics of mutation frequencies known as the Site Frequency Spectra (SFS), to compare the variant frequencies in DNA of sequenced HER2+ breast cancers, to those based on Model A and B simulations. The tumor-based SFS are better reproduced by Model A, pointing out a possible selection pattern of HER2+ tumor evolution. To put our models in context, we carried out an exploratory study of how publicly accessible data from breast, prostate, skin and ovarian cancers fit a range of models found in the literature.

## 1. INTRODUCTION

Determining the type of selection prevailing in evolving populations of cancer cells is still an open issue. There are many different models and views on the patterns of tumor evolution (McFarland et al., 2014; Sottoriva et al., 2015; Williams et al., 2016; McDonald et al., 2018; Dinh et al., 2020b; Tung and Durrett, 2021). In this paper, we plan to study clonal evolution patterns, produced by different mutation, drift and selection models. The topic is of current interest, as it is believed that deciphering the past of tumors leads to understanding of the causes of their growth and progression. We also hypothesize that inference from molecular data can be tied to the timeline of cancer progression before diagnosis, which is not observed. The outcome may impact the policies of early detection and prevention, which have public health importance.

Much of the modeling work is based on classical models of population genetics, generalized to accommodate time-varying cell population size. Reverse-time, genealogical, views of such models, commonly known as coalescent theory (Griffiths and Tavaré, 1998), have been used to infer aspects of the past of growing populations. Another approach is using branching processes, the simplest scenario being the linear birth-death process (lbdp), a binary fission Markov age-independent branching process (McDonald and Kimmel, 2017). A genealogical view of such models is also available (Lambert, 2008).

**FIGURE 1 |:**
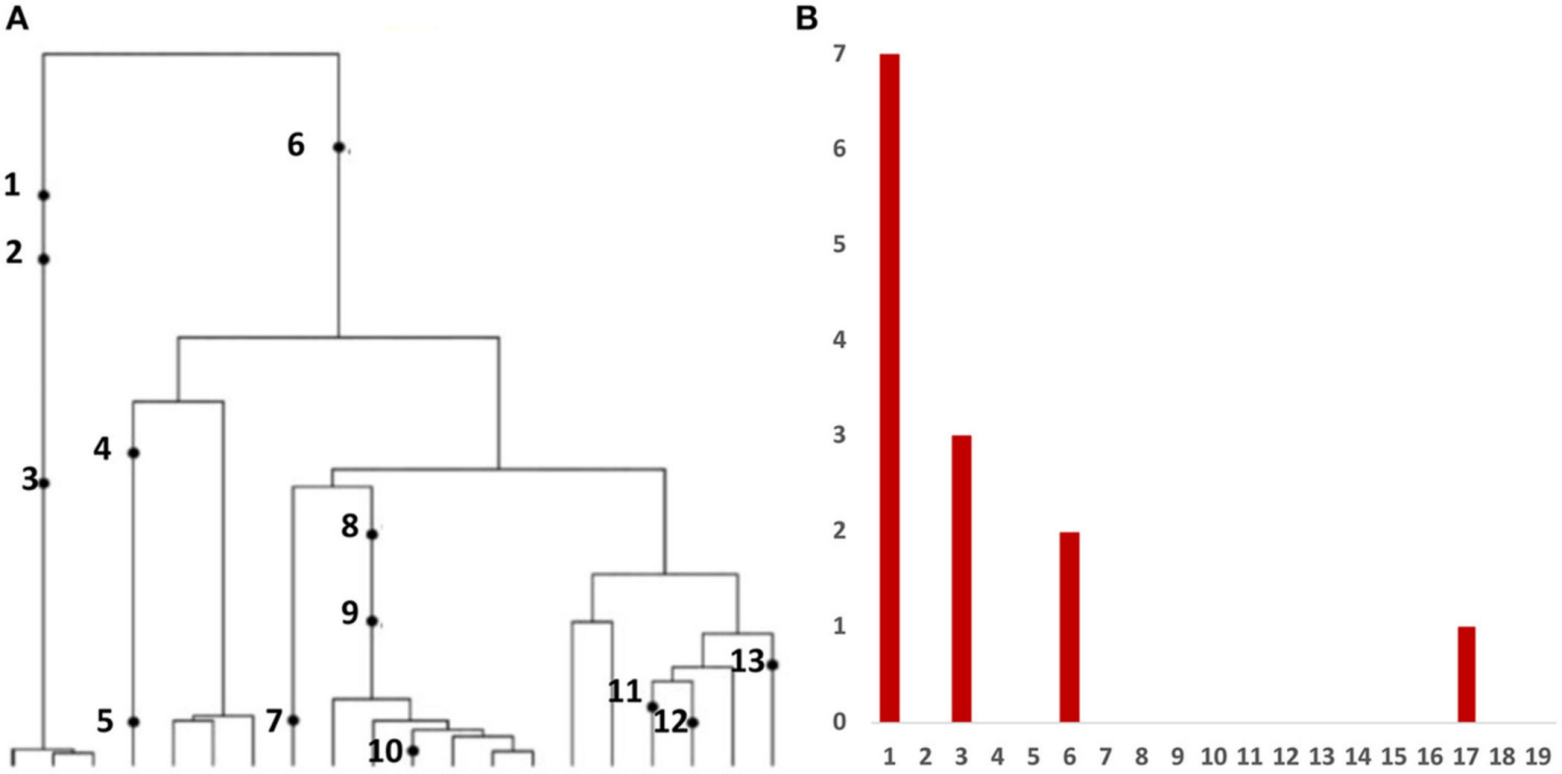
The Site Frequency Spectrum (SFS). **(A)** Genealogy of a sample of n=20 cells includes 13 mutational events, denoted by black dots. Mutations 4, 5, 7, 10, 11, 12, and 13 (total of 7 mutations) are present in a single cell, mutations 1, 2, and 3 (total of 3 mutations) are present in three cells, mutations 8 and 9 (2 mutations) are present in six cells, and mutation 6 (1 mutation) is present in 17 cells. **(B)** The observed site frequency spectrum, $S_{20}(1)=7$, $S_{20}(3)=3$, $S_{20}(6)=2$, and $S_{20}(17)=1$, other $S_{n}(k)$ equal to 0.

Variant allele frequency (VAF) spectrum, is the histogram of relative frequency of variant reads based on bulk sequencing of DNA extracted from tumor cells. This is the most frequent type of data affordable in large quantities. Inference from evolutionary models of DNA often exploits summary statistics of the sequence data, a common one being the so-called Site Frequency Spectrum (SFS), being an idealized version of VAF. In a sequencing experiment with a known number of sequences we can estimate, for each site at which a novel somatic mutation has arisen, the number of cells that carry that mutation. A very simple example is presented in Figure 1.

Cancer evolution is driven by two types of events: point mutations (and deletions/insertions) and copy number alterations, including major genomic rearrangements (Watkins et al., 2020). In bulk sequencing data, these events are reflected by changes in numbers of reference and variant reads. Existing mathematical and computational approaches include mostly techniques to estimate clusters of clones representing major genome transformation events and their evolution. We recently published a methodological paper (Dinh et al., 2020b) in which we provide a method of rigorously inferring parameters characterizing tumor evolution, based on analysis of site frequency spectra (SFS) computed using sequencing data from human tumors.

Our primary focus in the current paper is another approach (McFarland, 2014; McFarland et al., 2014, 2017), the tug-of-war model of evolution of cancer cell populations. The model became quite popular as a conceptual paradigm, explaining in an elegant manner the joint effect of rare advantageous and frequent slightly deleterious mutations, which may be identifiable with driver and passenger mutations in cancer (McFarland et al., 2017). Other approaches include a series of models by McDonald and Kimmel (2015) and McDonald et al. (2018), recently discussed among other by Cheek and Antal (2020) and Tung and Durrett (2021).

We will discuss two different versions of the tug-of-war model, both formulated as time-continuous Markov chains (TCMC). Both are phrased in the terms similar to the multitype Moran model. Moran model philosophy can be viewed as “competitive replacement,” by which individual cells face each other and inhibit each other’s right to be replaced by a direct descendant, under pressure from restrictive environment. This is opposed to the branching process “crowding out” in which a fastergrowing clone makes the slower-growing one rare to the extent of nonexistence. Historically, a version interpolating between the two approaches is the very influential Gerrish and Lenski model (Gerrish and Lenski, 1998). The original tug-of-war model is a state-dependent multitype branching process. We employ the constant-population Moran framework, to exploit the mutation-drift-selection interaction in a pure form. We will relate the process to the SFS of breast cancers and their lymph-node metastases.

As mentioned, we are among other interested in the testable differences between the so-called Darwinian and non-Darwinian mode of tumor evolution. We structure our models in such way that in one of the models (Model A) expected fitness in absence of mutations remains constant, while in the other (Model B) it is only non-decreasing.

We begin with mathematical definitions of the Moran model and branching process versions of tug-of-war. Then, in the Section 3, we present mathematical and simulation results, which demonstrate the differences between the long-term behavior of the two versions. We also use some typical population genetics non-neutrality tests to see how the effects of tug-of-war competition are reflected in testing. Finally, we match the site frequency spectra (SFS) obtained by simulation to the variant allele frequency spectra (VAF) obtained from sequencing of cancer DNA samples. Analysis will be based on the breast cancer data at our disposal as well as on data from The Cancer Genome Atlas (TCGA).

As pointed out by one the reviewers, for the last 10 years, asexual evolution including complex linkage effects, random mutation, and random genetic drift, has been described by the traveling wave theory; see Good et al. (2012), which includes a number of fundamental references. We also identified a recent paper (Grossmann et al., 2020) which concerns using the traveling waves theory to compare superdrivers to drivers in cancer models. Although our approach is based on discrete stochastic models, genealogies and direct simulations, linking it with traveling waves seems to be an interesting possibility.

## 2. MODELS AND DATA

### 2.1. Site Frequency Spectrum

As mentioned in the Introduction, in a sequencing experiment with a known number of sequences, we can estimate for each site at which a novel somatic mutation has arisen, the number of cells that carry that mutation. Inference from evolutionary models of DNA often exploits summary statistics of the sequence data, a common one being the so-called Site Frequency Spectrum. In a sequencing experiment with a known number of sequences, we can estimate for each site at which a novel somatic mutation has arisen, the number of cells that carry that mutation. These numbers are then grouped into sites that have the same number of copies of a mutant. Figure 1 gives an example; time is running down the page. The genealogy of a sample of n=20 cells includes 13 mutational events. We can see that mutations 4, 5, 7, 10, 11, 12, and 13 (a total of 7 mutations) are present in a single cell, mutations 1, 2, and 3 (total of 3 mutations) are present in 3 cells, mutations 8 and 9 (a total of 2 mutations) are present in six cells, and mutation 6 is present in 17 cells. If we denote the number of mutations present in k cells by $S_{n}(k)$, we see that in this example, $S_{n}(1)=7$, $S_{n}(3)=3$, $S_{n}(6)=2$, and $S_{n}(17)=1$, with all other $S_{n}(j)$ equal to 0. The vector $\left(S_{n}(1),S_{n}(2),\ldots ,S_{n}(n-1)\right)$ is called the (observed) Site Frequency Spectrum, abbreviated to SFS. It is conventional to include only sites that are segregating in the sample, that is, those for which the mutant type and the ancestral type are both present in the sample at that site. Mutations that occur prior to the most recent common ancestor of the sampled cells will be present in all cells in the sample; these are not segregating and are called truncal mutations.

In most cancer sequencing experiments, we do not know the number of cells that were sampled, and, indeed, the DNA sequence of each cell cannot be determined from bulk sequencing data. Nonetheless, we can estimate the relative proportion of the mutant at each segregating site, and so arrive at a frequency spectrum based on proportions. Accordingly, instead of writing $S_{n}(k)$, we write S(x)=S(k/n), with x treated as a continuous variable, such that x∈(0,1). We continue to use the term SFS for such a spectrum, as there should be no cause for confusion. In essence, S(x) is an idealized version of the empirical variant allele frequency (VAF) graph. In addition, it is convenient for reasons explained in Section 2.4 to define the cumulative tail of the SFS S(x)

(1) $$T(x)=\int_{x}^{1}S(\xi )\;d\xi ,\;x\in [0,1]$$

The theory that allows computing the expectations of SFS in populations with a given growth law under the Infinite Site Model (ISM) of mutation, was developed concurrently by many researchers, with one of the seminal papers published in 1998 by Griffiths and Tavaré (1998). The Griffiths-Tavaré expressions are accurate but quite complicated. A computational method which works fast even with very large sample sizes, was developed in a series of papers by Polanski and Kimmel (2003). Tractable approximations were derived under the exponential growth hypothesis by Durrett (2013). A related approach based on linear birth-and-death processes is that by Lambert (2008).

### 2.2. Tug-of-War Between Drivers and Passengers

We describe two versions of the Time-Continuous Markov Chain tug-of-war process, comparison of which relates to the question of Darwinian vs. non-Darwinian evolution in cancer. Both versions describe directional multiplicative selection. The parameters of the models are as follows:

- N population size (number of cells),
- μ mutation rate per cell,
- p probability that mutation is an advantageous driver, 1−p probability that mutation is a deleterious passenger,
- ${\left(1+s\right)}^{\alpha }{\left(1-d\right)}^{\beta }$ fitness of a cell with α driver mutations and β passenger mutations, where s is the selective advantage of the driver, and d is the selective disadvantage of the passenger
- the total rate of cell death at any given time, ${\text{Σ}}_{𝓟}$, equals the sum of the fitnesses of all cells in the population.

Further details concerning the transition rules in Models A and B are provided below.

#### 2.2.1. Model A

In this version of the model, we put the tug-of-war in the context of Moran model with multiple allelic types that differ with respect to selective value, which serves as mathematical framework for what can be viewed as “competitive replacement,” by which individual cells face each other and they compete with each other’s right to be replaced by a direct descendant.

We consider a population of a fixed number N of cells, each of them characterized by a pair of integers $\gamma_{i}=\left(\alpha_{i},\beta_{i}\right)$, corresponding to the numbers of drivers and passengers in its genotype, respectively. This pair determines the fitness $f_{i}$ of the i-th cell by the formula

(2) $$f_{i}=f_{i}\left(\alpha_{i},\beta_{i}\right)={\left(1+s\right)}^{\alpha_{i}}{\left(1-d\right)}^{\beta_{i}},\;i=1,\ldots ,N,$$

where s>0, the selective advantage of the driver and d∈(0,1) the selective disadvantage of the passenger, are parameters describing selective advantage of driver mutations over passenger mutations. These are called the selection coefficients, of driver and passenger mutations, respectively (see the Natural Selection chapter of the book by Durrett, 2008). The multiplicative form of the effect of multiple mutations is used in the population genetics literature, because it corresponds to lack of biological epistatic interaction; if one considers infinite population size, one can show that different sites evolve independently under this assumption (c.f., McFarland et al., 2014 for references).

There are two possible types of events: death - replacement and mutation. Under the time-continuous Markov Chain model, the times to nearest event are exponentially distributed. Briefly, exponential distributions form a 1-parameter family, with the parameter equal to the inverse of the expectation. The parameter of the exponentially distributed time to the next death replacement event is equal to ${\text{Σ}}_{𝓟}=\sum_{f_{i}\in 𝓟}f_{i}$, where 𝓟 is the set of fitnesses of cells present before the death - replacement event. We assume that the dying cell i is drawn from distribution biased by fitness, i.e., with probability mass function (pmf) $\{f_{i}/{\text{Σ}}_{𝓟},f_{i}\in 𝓟\}$. In addition, the replacing cell j is also drawn from distribution biased by fitness, with pmf $\left\{f_{j}/{\text{Σ}}_{𝓟},f_{j}\in 𝓟\right\}$. The end state may be the same as the starting state (the replacing cell may be the same as the dying cell).

The parameter of the independently distributed exponential time to the next mutation is equal to Nμ, where μ is the mutation rate per cell. The cell, chosen with probability 1/N, undergoes a mutation event, changing its state to either (α+1,β) or (α,β+1) with (conditional) probabilities p∈(0,1) and q=1−p, respectively.

In summary, the time to the next event is random and exponentially distributed with parameter

(3) $${\text{Σ}}_{𝓟}+N\mu$$

called the total rate of death - replacement and mutation events.

**FIGURE 2 |:**
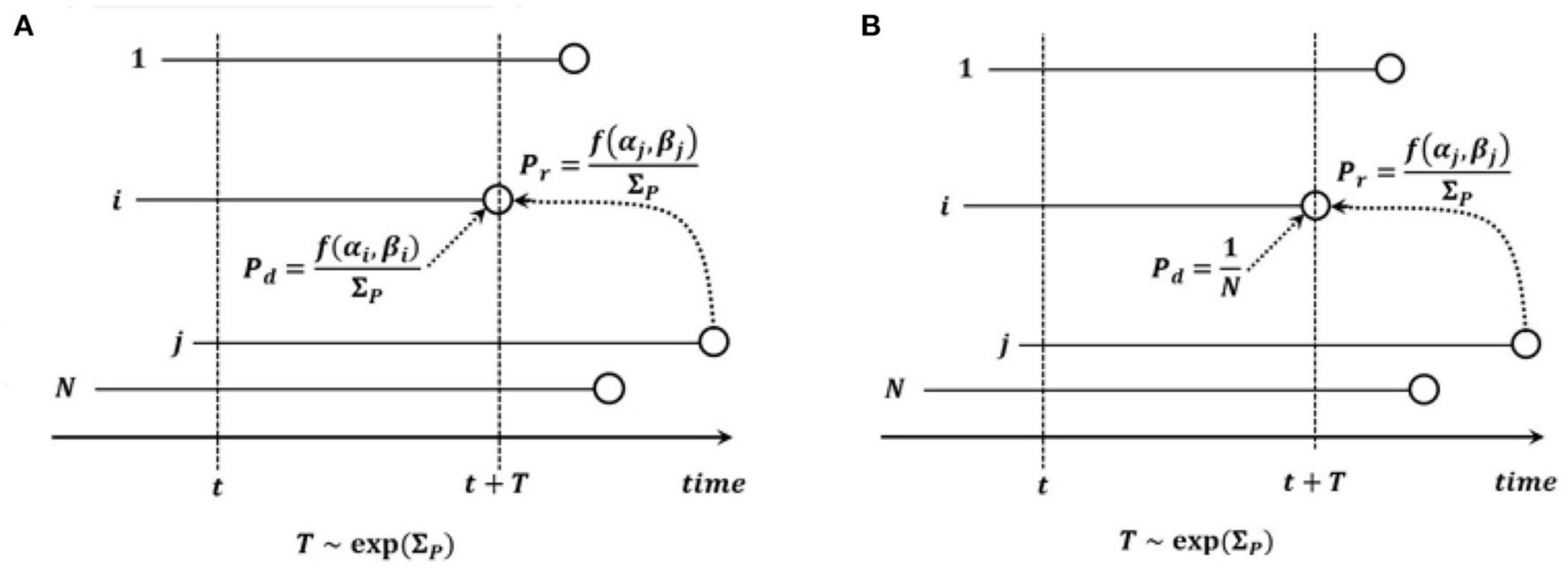
Graphical depiction of (**A**) Model A and (**B**) Model B. *Notation:* N, count of cells in the process; i, cell dying and to be replaced; j, cell replacing cell i; t, current time; T, time to death - replacement event; f(α,β), fitness of cell with α drivers and β passengers; ${\text{Σ}}_{P}={\text{Σ}}_{i}f\left(\alpha_{k},\beta_{k}\right)$.

Model B in the version we consider here, is defined similarly to Model A, with the parameter of the exponentially distributed time to death - replacement being equal to ${\text{Σ}}_{𝓟}$, but the dying cell i is drawn from a uniform distribution on all the N cells before death - replacement (see Figure 2). We allow the possibility that the end state may be the same as the starting state (the replacing cell may be the same as the dying cell). In the original formulation of Model B in Bobrowski et al. (2021), this possibility was excluded, which lead to notational differences. In this model, the time to the next event is random and exponentially distributed with the parameter the same as in Equation (3).

#### 2.2.2. Model A vs. Model B

The most important difference is that of the expected value of fitness increment in the population at the moment of death - replacement in Model A vs. Model B. The fitness increment is equal to the difference $f_{j}-f_{i}$, where $f_{i},f_{j}$ are fitnesses of the dead cell and of the new cell, in the absence of mutations. The expected fitness change for Model A is equal to

(4) $$\text{Δ}f_{A}=\frac{\sum \sum_{f_{i},f_{j}\in 𝓟}f_{i}f_{j}\left(f_{j}-f_{i}\right)/{\text{Σ}}_{𝓟}}{{\text{Λ}}_{A}}=C_{A}\sum_{f_{i},f_{j}\in 𝓟}f_{i}f_{j}\left(f_{j}-f_{i}\right)=0$$

where $C_{A}$ is a constant.

However, the expected fitness change for Model B is equal to

(5) $$\text{Δ}f_{B}=\frac{\sum \sum_{f_{i},f_{j}\in 𝓟}f_{j}\left(f_{j}-f_{i}\right)/{\text{Σ}}_{𝓟}}{{\text{Λ}}_{B}}=C_{B}\underset{f_{i}\neq f_{j}\in 𝓟}{\sum \sum }f_{j}\left(f_{j}-f_{i}\right)\;\;=\frac{C_{B}}{2}\underset{f_{i}\neq f_{j}\in 𝓟}{\sum \sum }{\left(f_{i}-f_{j}\right)}^{2}\geq 0$$

where $C_{B}$ is a constant. $\text{Δ}f_{B}=0$ if and only if all N cells have the same fitness.

As a conclusion, trends in trajectories of Model A are expected to depend only on the balance of drivers and passengers. The trends in Model B are more complex, as explained in Bobrowski et al. (2021). The drift and selection pattern in Model B biases it toward increasing fitness.

#### 2.2.3. Trends of Expected Fitness in the Mutation Process

As mentioned earlier on, μ is the mutation rate. As a result of mutation, the cell changes state to either (α+1,β) (driver mutation) or (α,β+1) (passenger mutation) with probabilities p∈(0,1) and q=1−p, respectively.

As noted by Bobrowski et al. (2021), the equilibrium condition for no change of the expected fitness change resulting from a mutation, has the form

(6) ps=(1−p)d

for both models. As a result, we obtain the expected fitness unchanged by a mutation event if ps=(1−p)d, increasing if ps>(1−p)d and decreasing if ps<(1−p)d. For Model A, in which the death - replacement process leaves the expected fitness intact, the expected fitness trend follows the mutation process trend. In Model B, the outcome is more complex, as explained mathematically in Bobrowski et al. (2021) and using simulations, further on.

### 2.3. Other Models

#### 2.3.1. Site Frequency Spectra Under Neutrality and Exponential Growth

Griffiths and Tavaré (1998) provide a general coalescent framework for the expected number $ES_{n}(k)$ of mutant sites having k copies of the mutant in a sample of size n, drawn from a Wright-Fisher population model with size changing deterministically in the past, under the Infinite Sites Model (ISM). Among other, they showed that

(7) $$ES_{n}(k)=\theta \sum_{j=2}^{n-k+1}jp_{nj}(k)ET_{j},$$

where

(8) $$p_{nj}(k)=\left(\begin{array}{c} n-k-1\\ j-2 \end{array}\right)/\left(\begin{array}{c} n-1\\ j-1 \end{array}\right),$$

the $T_{j}$ denoting the coalescence times for the model with arbitrary functional form of growth or decline of the population in the past. The expectations are generally difficult to derive analytically, and therefore it is convenient to consider the approximations provided by Durrett (2013), who showed that if the population has been growing exponentially with growth rate r, i.e., $N(t)=Ne^{rt}$, t<0, where N is the present population size, then as N→∞,

(9) $$ES_{n}(k)\to \frac{\theta }{r}\frac{n}{k(k-1)},k=2,\ldots ,n-1,$$

while

(10) $$ES_{n}(1)∼\frac{\theta n\ln(rN)}{r},$$

where ∼ denotes asymptotic equivalence. This latter term follows directly from Griffiths and Tavaré (1998) results.

Relevance of the singletons for cell DNA sequencing data is questioned by many, since low-frequency variants are routinely pruned by data-cleaning algorithms to avoid confusion with sequencing errors. We discuss this question further on. Concerning non-singletons, i.e., doublets, triplets, and so forth, expression (9) implies that the total count of these mutations is equal to

(11) $$A=\sum_{k=2}^{n-1}ES_{n}(k)\approx \sum_{k=2}^{n-1}\frac{\theta }{r}\frac{n}{k(k-1)}=n\frac{\theta }{r}\left(1-\frac{1}{n-1}\right)\approx n\frac{\theta }{r}$$

**FIGURE 3 |:**
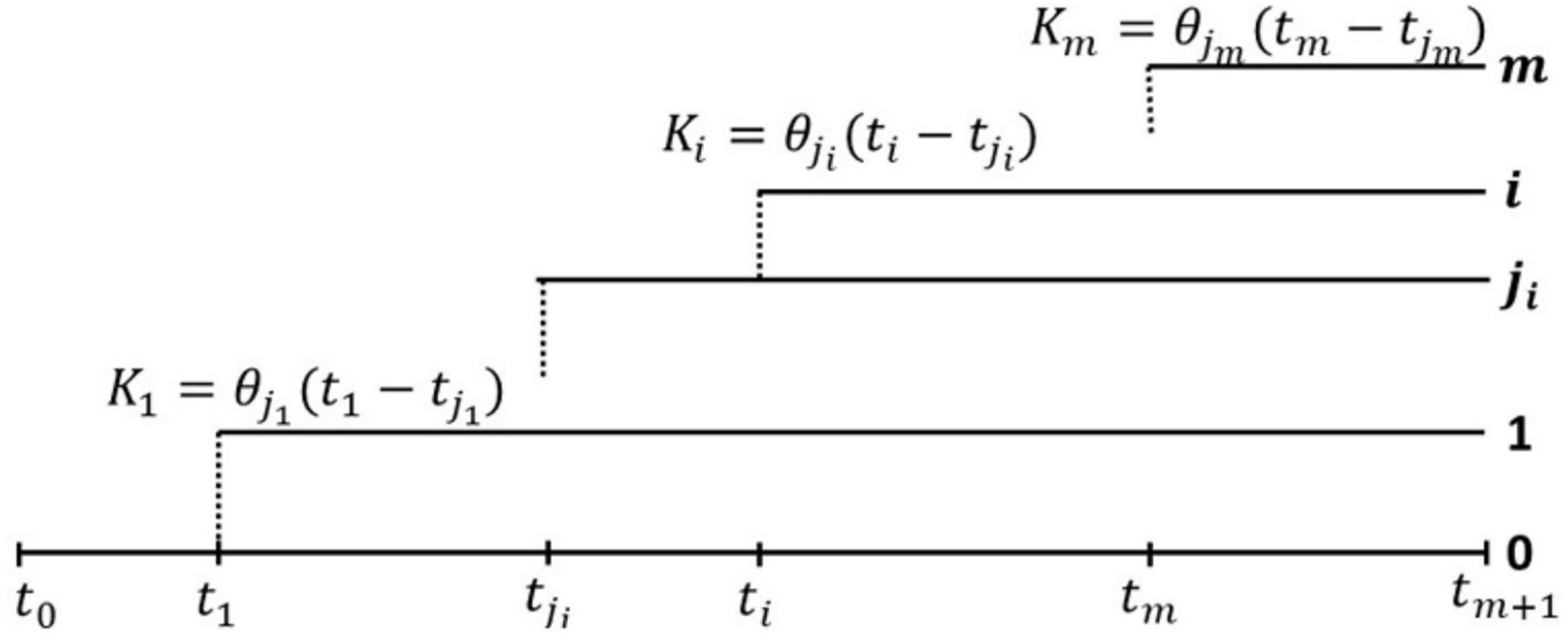
General clonal hierarchy of tumor cells in the model of Dinh et al. (2020b). At time $t_{i}$, Clone i, 1≤i≤m, branches off from Clone $j_{i}$, where $0\leq j_{i}\leq m-1$, and $j_{i}<i$, as depicted in figure. Let us note that $j_{1}=0$, and for completeness we assume $j_{0}=0$. Moreover we assume that cells in each Clone i mutate (neutrally) according to the infinite sites model (ISM), i.e., each mutation occurs at a different genome site, with mutation rates $\theta_{i}$ per time unit per cell, and Clone i grows exponentially at rate $r_{i}$ (Equation 12). At time $t_{m+1}$, the tumor is diagnosed and the cell count at this time is denoted N. Finally, we assume that each clone is initiated by a single cell of clone ji, the ancestry of which is marked by $k_{i}$ mutations in Clone ji (Equation 13).

Operationally, expressions (9), (10), and (11) are the simplest to use. Since our simulations will be performed using the linear birth-death processes and not Wright-Fisher model with exponential growth, we should in principle use the corresponding SFS expressions, such as those derived in Appendix E to Dinh et al. (2020b). However, these latter involve Gauss hypergeometric functions and, numerically, they work very much like Durrett’s approximations (see Dinh et al., 2020b; Figure 3).

#### 2.3.2. Neutral Evolution With Episodic Selective Sweeps

A model of tumor evolution can be based on competition of clones with differential growth rates, which gradually replace (sweep out) each other. The selective sweeps are initiated by major “driver” events such as genome rearrangements or cancer gene mutations. They are separated by neutral mutations not affecting growth rates, but merely being ticks of a molecular clock. In our recent paper (Dinh et al., 2020b), we developed a sampling theory for such model. As we will see, the subsequent genome clones will be reflected by humps superimposed on the Griffiths-Tavaré neutral SFS.

In general, we assume a general clonal hierarchy, in which at time $t_{i}$, Clone i,1≤i≤m, branches off from Clone $j_{i}$, where $0\leq j_{i}\leq m-1$, and $j_{i}<i$, as depicted in Figure 3. Let us note that $j_{1}=0$, and for completeness we assume $j_{0}=0$. The clonal structure can be summarized by an (m+1,m+1) clonal hierarchy matrix B, which has the 0-th row filled with 0−s, and each subsequent i-th row, i=1,…,m also filled with 0−s, except for the $j_{i}$-th column, where the entry equals 1.

Moreover, we assume that cells in each Clone i mutate (neutrally) according to the infinite sites model (ISM), i.e., each mutation occurs at a different genome site, with mutation rates $\theta_{i}$ per time unit per cell, and Clone i grows exponentially at rate $r_{i}$. At time $t_{m+1}$, the tumor is diagnosed and the cell count at this time is denoted N. Therefore, the fraction of cells of any Clone i, present at $t_{m+1}$, is equal to

(12) $$p_{i}=\frac{e^{r_{i}\left(t_{m+1}-t_{i}\right)}}{\sum_{l=0}^{m}e^{r_{i}\left(t_{m+1}-t_{l}\right)}}=\frac{e^{r_{i}\left(t_{m+1}-t_{i}\right)}}{N},\;i=0,\ldots ,m$$

Finally, we assume that each clone is initiated by a single cell of clone $j_{i}$, the ancestry of which is marked by $K_{i}$ mutations in Clone $j_{i}$, which are just measuring time to the genomic (or other) event leading to the rise of Clone i. Alternatively, the last of these mutations may be “the event” itself. In the expected value sense, this leads to

(13) $$K_{i}=\theta_{j_{i}}\left(t_{i}-t_{j_{i}}\right),\;i=1,\ldots ,m$$

as depicted in Figure 3. In each clone accumulation of the neutral mutations follows the rules of the infinite sites model (ISM), with the resulting SFS similar as in Equation (9), specifically

(14) $$ES_{n}=\frac{\theta_{i}}{r_{i}}\frac{n}{k(k-1)},\;i=0,\ldots ,m$$

##### Two-clone toy example

**FIGURE 4 |:**
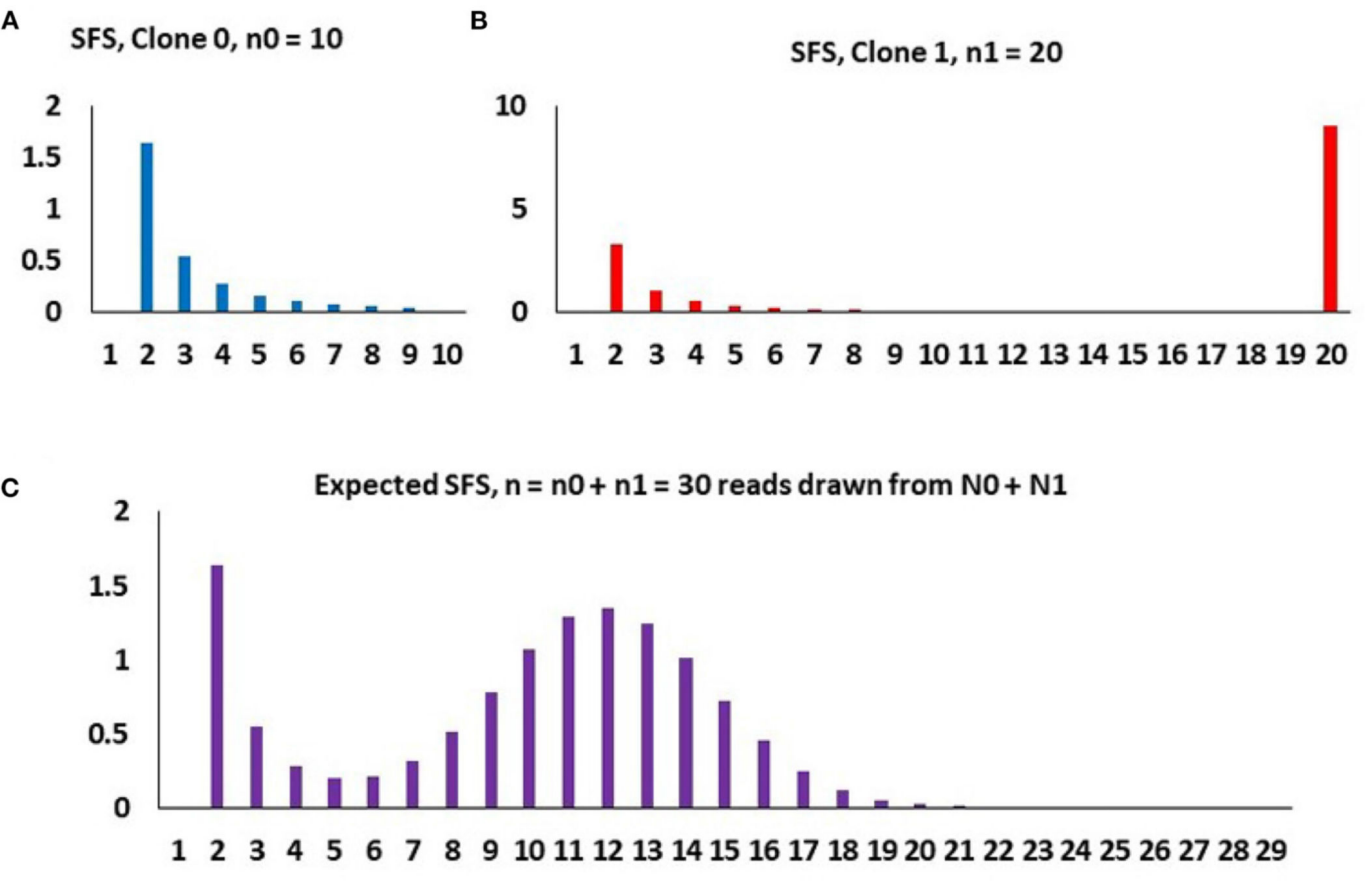
An example of how the composite SFS for two clones (m=1) arises under binomial sampling of DNA reads, with parameters, n=30, $K_{1}=8$, $p_{1}=0.4$, and A=3.4. **(A)** Purely neutral SFS of Clone 0 based on the sample of $n_{0}=10$ cells, mathematically $q_{k}^{0}=p_{0}n_{0}\theta_{0}/\left[r_{0}k(k-1)\right]$, $k=2,\ldots ,n_{0}-1$. **(B)** SFS of Clone 1 based on the sample of $n_{1}=20$ cells, with a spike representing all 20 cell having the $K_{1}=8$ mutations defining Clone 1 and the neutral component, mathematically $q_{k}^{1}=p_{1}n_{1}\theta_{1}/\left[r_{1}k(k-1)\right]1\left(k<n_{1}\right)+K_{1}\delta_{k,n_{1}}$, $k=2,\ldots ,n_{1}$. **(C)** Spectrum of the entire population, based on randomly sampled DNA reads from n cells, mathematically $Q_{nk}$, as in expressions (Equations 17, 18). The binomial hump is due to random sampling of different counts of Clone 0 and Clone 1 DNA reads, independently for each mutation, yielding $n_{1}∼\text{binomial}\left(n,p_{1}\right)$.

To understand how the model works, let us use a two-clone toy example as depicted in Figure 4. Assume that at time $t_{0}=0$, the initial malignant cell population (Clone 0) arises, grows exponentially in size at rate $r_{0}$, these cells acquiring mutations at the rate $\theta_{0}$ per time unit per cell. At time $t_{1}>0$, a secondary clone (Clone 1) arises, which differs from the original clone with respect to growth rate (now equal to $r_{1}$) and mutation rate (now equal to $\theta_{1}$). We call this the “selective event.” The new clone arises on the background of a haplotype already harboring $K_{1}$ mutations. Finally, at $t_{2}>t_{1}>0$, the sample of DNA is made available for sequencing. At that point, it is difficult to distinguish cells arising from the two (or more) clones and the resulting sample represents a mixture of DNA from both.

We assume that both clones start from single cells, so that the sequenced sample comes from $N=N_{0}+N_{1}$ cells, and the number of cells in each clone is

(15) $$N_{0}=\exp\left(r_{0}t_{2}\right),\;N_{1}=\exp\left(r_{1}\left(t_{2}-t_{1}\right)\right),$$

and the fraction of clone i cells is approximately equal to

(16) $$p_{i}=N_{i}/\left(N_{0}+N_{1}\right),\;i=0,1.$$

Based on this, we use the neutral Griffiths-Tavareé SFS under exponential growth to estimate the expected site frequency spectra. We obtain the following expression for the expected count of variants present in k copies in the sample of n cells

(17) $$Q_{nk}=ES_{n}(k)=\frac{A}{k(k-1)}+K\left(\begin{array}{l} n\\ k \end{array}\right)p_{0}^{n-k}p_{1}^{k}$$

for k=2,…,n, where

(18) $$A=n\left(\frac{p_{0}\theta_{0}}{r_{0}}+\frac{p_{1}\theta_{1}}{r_{1}}\right)$$

and notation $Q_{nk}$ has been retained for consistency with Dinh et al. (2020b). The final take-out message from the toy example is that (i) the total count of neutral non-singleton mutations in both clones is equal to A, the area under the decreasing rights-skew component of the SFS, (ii) the total count of mutations in the lineage leading to emergence of Clone 1 is equal to K, the area under the binomial hump of the SFS, and (iii) the fraction of the Clone 1 cells is equal to the central parameter of the hump of the SFS. Parameters A, K and $p_{1}$ are directly estimable from the SFS.

##### Back to general clonal hierarchies

It is possible to extend (Equation 17) to arbitrary number of clones. If the last clone formed has index m, then the more general expected SFS equation has the form

(19) $$Q_{nk}=ES_{n}(k)=\sum_{i=0}^{m}\frac{p_{i}A_{i}}{k(k-1)}+\sum_{i=1}^{m}K_{i}\left(\begin{array}{l} n\\ k \end{array}\right){\left(1-P_{i}\right)}^{n-k}P_{i}^{k},\;k=2,\ldots ,n-1$$

where the form of $A_{i}$ is deduced from Equation (14). The relations between the centroid $P_{i}$ of the i−th binomial hump and the fraction $p_{i}$ of cells in the i−th clone results from the fact that each clone’s binomial hump in the composite SFS arises from the $K_{l}$ mutation counts from all Clones l preceding clone i. We skip the resulting algebra here.

#### 2.3.3. Birth and Death Process With Mutations

Mathematical and simulation treatment of a model, introduced by McDonald et al. (2018), was recently published by Tung and Durrett (2021).

In the model we consider, clonal expansion begins with a single cell of the original tumor-initiating type (type 0). Type 0 individuals give birth at rate $a_{0}$ and die at rate $b_{0}$, so the exponential growth rate is $\lambda_{0}=a_{0}-b_{0}$. For simplicity, we will suppose that neutral mutations accumulate during the individual’s life time at rate ν, instead of only at birth.

Type 0 individuals mutate to type 1 at rate $u_{1}$. Type 1 individuals give birth at rate $a_{1}$ and die at rate $b_{1}$. Their exponential growth rate is $\lambda_{1}=a_{1}-b_{1}$ where $\lambda_{1}>\lambda_{0}$. All type 1 mutations have the same growth rate.

Tung and Durrett (2021) demonstrate that if the fitnesses of the two types are $\lambda_{0}<\lambda_{1}$ then the site frequency spectrum has the form of $cf^{-\alpha }$ where $\alpha =\lambda_{0}/\lambda_{1}$. Again, this is a power-law SFS, due to the advantageous mutations that produce the founders of the type 1 population. Mutations within the growing type 0 and type 1 populations follow the 1/f law. Authors assert that the results show that neutral evolution can be distinguished from the two-type model using the site frequency spectrum.

### 2.4. Comparison of SFS Tails in Different Models

**FIGURE 5 |:**
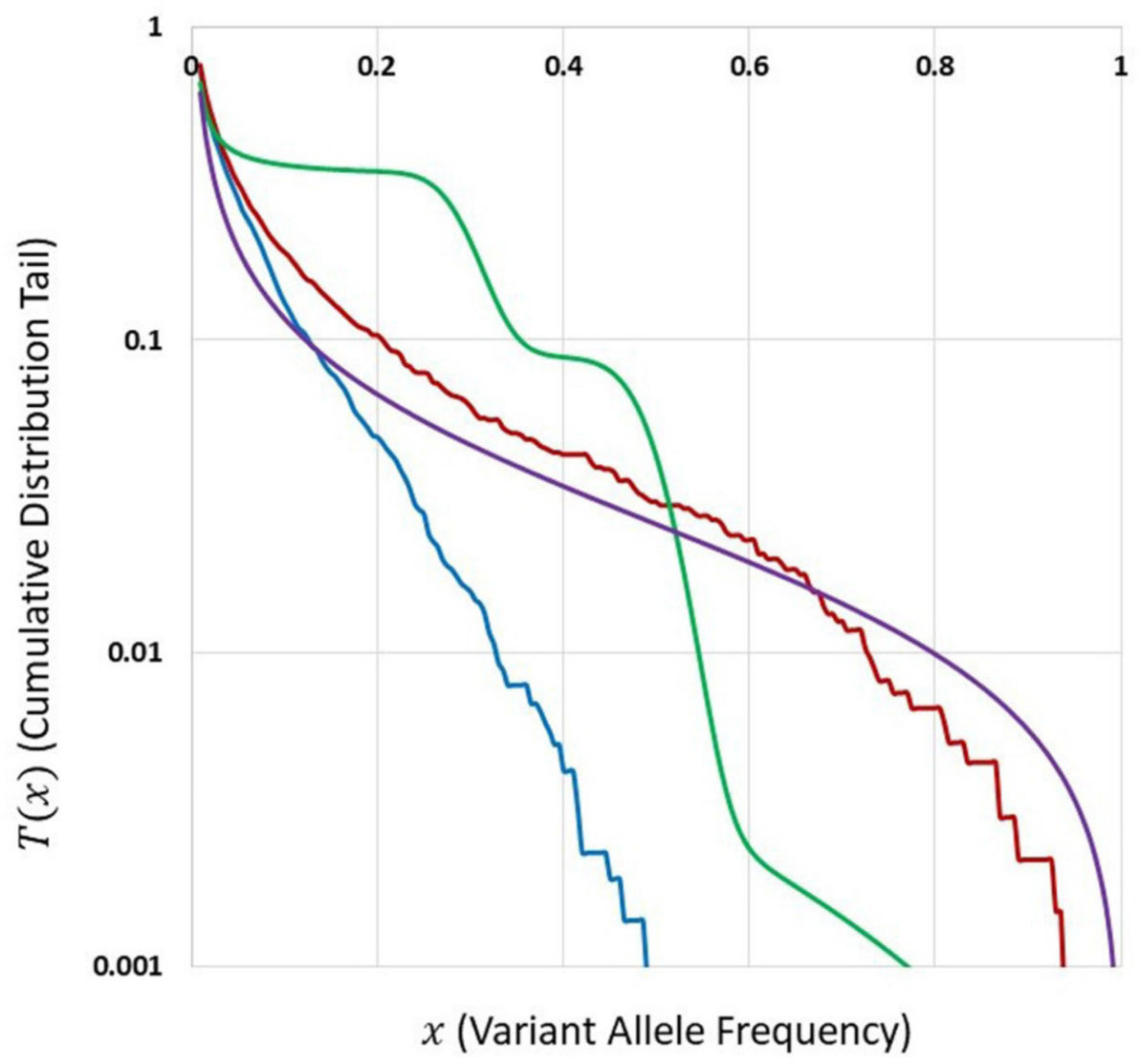
Comparison of cumulative tails in semi-logarithmic coordinates yielded by different theoretical models of cell proliferation, mutation and selection. *Blue:* Model A. *Red:* Model B. Both with identical parameters s=0.5, d=0.0001, L=6, p=0.01, t=100, and N=200. *Green:* Multiclone model of Dinh et al. (2020b), with parameters m=3, n=200, A=40, $K_{1}=5$, $K_{2}=15$, $P_{1}=0.5$, and $P_{2}=0.3$. *Purple:* Model of McDonald et al. (2018), based on a smoothed version of a Figure in Tung and Durrett (2021).

Figure 5 depicts cumulative tails of the SFS in semi-logarithmic coordinates, yielded by different theoretical models of cell proliferation, mutation and selection. Comparison of cumulative tails, as opposed to probability mass functions or probability distribution functions, seems meaningful for several reasons; (i) the cumulative tails are smoother, (ii) they are all inscribed into a unit square, if VAF frequency x=m/n is used as an argument, (iii) semi-logarithmic coordinates allow resolving differences in the “deep tail” (T(x) small), and (iv) they are less sensitive to differences in T(x) for x small, which might be caused by DNA sequencing errors and data “massaging.” Blue and red curves correspond to Models A and B with parameters for which Model A seems to be fitting our breast cancer data best (see Results). The relationships between Models A and B SFS, is investigated further on. Multiclone model of Dinh et al. (2020b) leads to a tail that is characteristic of such cancers as advanced ovarian cancer or melanoma (see further on). McDonald et al. (2018) model tail seems to be similar to Model B, but Model B exhibits a range of patterns (see Results).

### 2.5. Neutrality Testing

The hypothesis of selective neutrality, leading to the “neutral” theory of evolution, is credited to Kimura (1968). The theory assumes that the number of mutations that have occurred by random stochastic processes without selective impact, strongly exceeds the number of mutations affected by selection. The aim of neutrality testing is to determine whether the observed alelle counts $a_{1},\ldots ,a_{n}$ conform to what is expected under null hypothesis assuming neutrality, given the sample size n and the observed number k of alleles in the sample.

Accordingly, in the present analysis, we use counting rules such that each new mutation is creating a new allele in the individual cell, i.e., using the infinite allele model (IAM). This allows using neutrality tests based on the Ewens Sampling Formula.

#### 2.5.1. Expected Allele Count

The properties of a sample of n genes under infinitely many alleles version of the Wright-Fisher model are best summarized through the following (approximating) partition formula. Let us define $A=\left(A_{1},A_{2},\ldots ,A_{n}\right)$ where $A_{i}$ if the number of alleles present in exactly $a_{j}$ cells (out of n) in the sample. With this definition, the following expression, the well-known Ewens Sampling Formula (ESF) was derived by Ewens (1972) and Karlin and McGregor (Karlin, 1972). It describes the distribution of selectively neutral alleles under mutation-drift equilibrium, and under the infinite allele model.

(20) $$\mathbb{P}(A=a)=\frac{n!\theta \sum a_{j}}{1^{a_{1}}2^{a_{2}}\ldots n^{a_{n}}a_{1}!a_{2}!\ldots a_{n}!S_{n}(\theta )},$$

where $a=\left(a_{1},a_{2},\ldots ,a_{n}\right)$ and $S_{n}(\theta )$ is defined by

(21) $$S_{n}(\theta )=\theta (\theta +1)(\theta +2)\ldots (\theta +n-1)$$

where θ is the scaled mutation rate (see next praragraph). Let us denote $\sum A_{j}$, the (random) number of different allelic types seen in the sample, by K, and $\sum a_{j}$, the corresponding observed number in a given sample, by k. We have $\sum jA_{j}=\sum ja_{j}=n$. From Equation (20) the probability distribution of the random variable K can be obtained as

(22) $$\text{ℙ}(K=k)=\left|S_{n}^{k}\right|\theta^{k}/S_{n}(\theta )$$

In our case, n=N, and θ=nμ/λ, where μ denotes mutation rate and λ corresponds to allele fitness (in the neutral case fitness is always equal to 1). Fitness correction is added to account for cell generation length in the model, which is inversely proportional to fitness. Quantity $\left|S_{n}^{k}\right|$ is the coefficient of $\theta^{k}$ in $S_{n}(\theta )$ and is calculated as the absolute value of a Stirling number of the first kind. Both these symbols result from mathematical derivations and do not seem to have a direct biological interpretation. Expression (22) with the mutation rate θ known provides the distribution under null hypothesis of neutrality i.e., the hypothesis that the alleles in the sample are selectively equivalent. Empirical distribution under alternative hypothesis of Model A or B with different s and d coefficients can be obtained by running the model a large number of times and obtaining frequencies of alleles present in given number of cells. Then the empirical distribution can be compared to the analytically expressed null, using a goodness of fit test such as the one-sample Kolmogorov-Smirnov (K-S) test.

#### 2.5.2. Expected Singleton Count

In this case the testing procedure is based on the sample frequency spectrum. Let us again define $A_{j}$ as the random count of alleles in the sample that are represented by exactly j genes. For given k and n the mean value of $A_{j}$ can be found directly as

(23) $$E\left(A_{j}|k,n\right)=\frac{n!}{j(n-j)!}\frac{\left|S_{k-1}^{n-j}\right|}{\left|S_{k}^{n}\right|}$$

In this expression, the $S_{j}^{i}$ are values of Stirling numbers of the first kind, the array of the $E\left(A_{j}|k,n\right)$ values for j=1,2,…,n is the sample conditional mean frequency spectrum, and the corresponding array of observed values $a_{j}$ is the observed conditional frequency spectrum. The j=1 term in both these vectors is singleton count.

The singleton distribution under neutrality (null hypothesis) is approximated by substituting into expression (23), with j=1, the empirical k from each simulation run, thus obtaining the conditional expectation of singleton count given K=k, and computing the empirical distribution of these expectations. This latter is then compared to the empirical distribution of singleton count from all runs of the model. For this purpose we use the two-sample two-sided Wilcoxon test, which is particularly sensitive to differences of central tendencies such as means or medians, but less so to differences in shape. Two-sample tests are justified by the semi-empirical nature of the null distribution.

### 2.6. DNA Sequencing of Cell Samples From Breast Cancer Specimens

#### 2.6.1. DNA Sample Collection and Processing

Paired tissue samples from primary breast tumor locations and concurrent metastasis to regional lymph nodes were collected at the Department of Applied Radiology of the Maria Sklodowska-Curie National Research Institute of Oncology, Krakow Branch (Poland). Cancer specimens were matched with specimens of normal tissue used as a reference for individual genetic background (control samples). Two sets of 3 samples each, called specimens G30 and G31, are HER2+ breast cancers. DNA samples were isolated at the Department of Applied Radiobiology from macro-dissected FFPE tissue specimens, processed to generate DNA libraries and sequenced using Illumina HiSeq platform (with min. 100x coverage).

Quality control whole exome sequencing (WES) experiment was conducted using FastQC and FastQ Screen. Raw reads were aligned to the GRCh38 reference genome using the BWA mem (v0.7.17) (Li, 2013) in the alternative contigs-aware mode. All aligned reads were processed using MarkDuplicates algorithm from the Picard tool set and BaseRecalibrator which is a part of the Genome Analysis Toolkit (GATK v4.1.4.0) (DePristo et al., 2011). Somatic mutations were identified using MuTect2 (v4.1.4.0) (DePristo et al., 2011) using tumor-normal sample pairs. Variants were filtered using GATK’s FilterMutectCalls based on MuTect2 results, as well as sample contamination estimates obtained using CalculateContamination tool and read orientation bias statistics obtained with LearnReadOrientationModel tool. All retained variants were annotated using the Variant Effect Predictor (v100) (McLaren et al., 2016). Further details concerning the quality control issues and a comparison between FFPE vs. FF (fast-frozen) DNA quality, are presented in the Part 3 of the Supplementary Materials.

#### 2.6.2. Removal of Coverage Difference Bias

In the experimental dataset, differences of coverage of variant sites by sequencing reads are present, which might bias the estimation of variant allele frequencies of mutations present both in the primary and in lymph node metastases. In order to correct for this effect, a total count histogram equalization method was developed. The method is based on resampling and it helps to minimize the effect of variation of total number of reads between the primary tumor sample and lymph node sample on the shape of their respective Site Frequency Spectra. The correction it yields is not large but seems noteworthy. We proceed as follows.

1. Histograms of total read counts are generated for both samples of the same individual. It is necessary to employ a common bin width for both histograms.
2. For each bin, the lower count among the two histograms ischosen as the new desired count for an equalized histogram.
3. The variants are sorted by total count of reads separately for lymph node sample and primary tumor sample. Sorted variants are divided based on the total count of reads into subsets corresponding to bins of the desired histogram.
4. For each bin, the corresponding subset with greater numberof variants (lymph node or primary tumor) is pruned by randomly choosing the desired number of variants.

## 3. RESULTS

Simulations presented in the Results section were performed with N ranging from 50 to 400 cells. This range of N allows carrying out direct simulations in manageable time. We devote part of Discussion section to biological interpretation of parameters.

### 3.1. Simulation Studies of Models A and B

Simulation results illustrating the behavior of the A and B models are ordered according to the fitness trend predicted by Equation (6). All simulations presented in this section were performed on 100 cells. The duration of simulation was generally equal to t=100, except in the case of high driver influence (sp>dq), where it was reduced to t=60 due to the high memory demand.

#### 3.1.1. Models A and B Without Driver and Passenger Impact, s=d=0

In the absence of driver and passenger impact (s=d=0) fitness of all cells is constant and equal to the initial value. To check how selection and drift processes impact the fitness of the population we consider the scenario in which there is lack of mutation process, but initial numbers of drivers and passengers affecting initial fitness are drawn from exponential distribution with parameters 10×p and 10×(1−p), respectively and rounded to the nearest integer.

**FIGURE 6 |:**
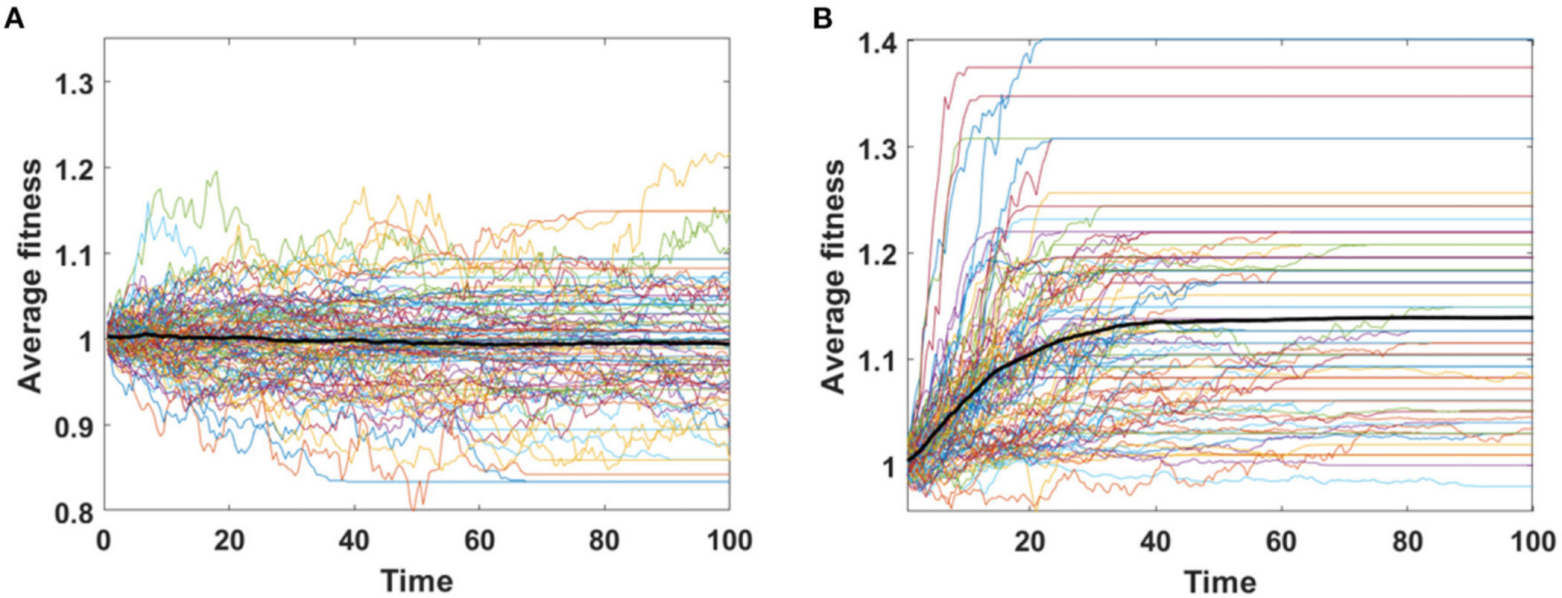
Average fitness of cells as a function of time. Results for 100 simulations involving N=100 cells with no mutation, but with initial number of drivers and passengers drawn from exponential distribution with parameter 10×p for number of drivers and 10×(1−p) for passengers, with p=0.5 and rounded to the nearest integer. (**A**) Model A; (**B**) Model B. Bold lines represent means of average fitnesses from 100 simulations.

Trajectories of average fitness for 100 simulations are presented in Figure 6. Mean fitness of 100 simulations remains almost constant and equal to 1 in Model A (Figure 6A), as expected by Equation (6), while in Model B increase in fitness is observed due to replacement of dying cells by the fitter ones (Figure 6B).

**FIGURE 7 |:**
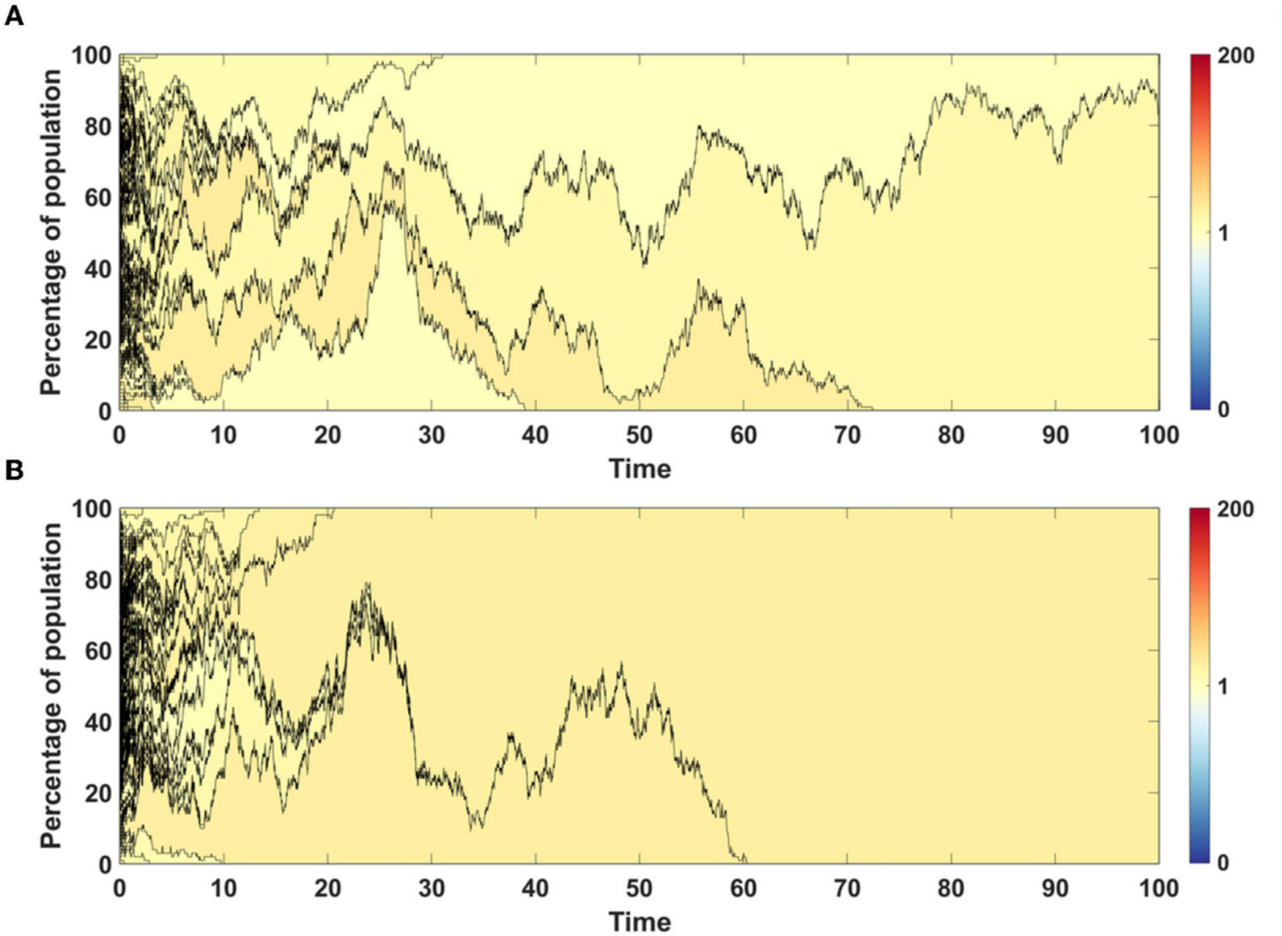
Time succession patterns of clones colored according to their fitness. Results of one simulation on N=100 cells with no mutation process, but with initial number of drivers and passengers drawn from exponential distribution with parameter 10×p for number of drivers and 10×(1−p) for passengers, with p=0.5. (**A**) Model A; (**B**) Model B.

For both models we chose single simulations to show changes in fitness and percentage of given clone in population. In both cases, in initial phase of the simulation less fit clones are purged from population and replaced by a few clones with higher fitness (Figures 7A,B).

#### 3.1.2. Symmetric, sp=d(1−p), Case of Models A and B

The following examples include the process of mutation. In all simulations the initial population is homogeneous with no driver or passenger mutations and with fitness of all cells equal to 1. Observed trends in average fitness depend on mutation and death - replacement events.

**FIGURE 8 |:**
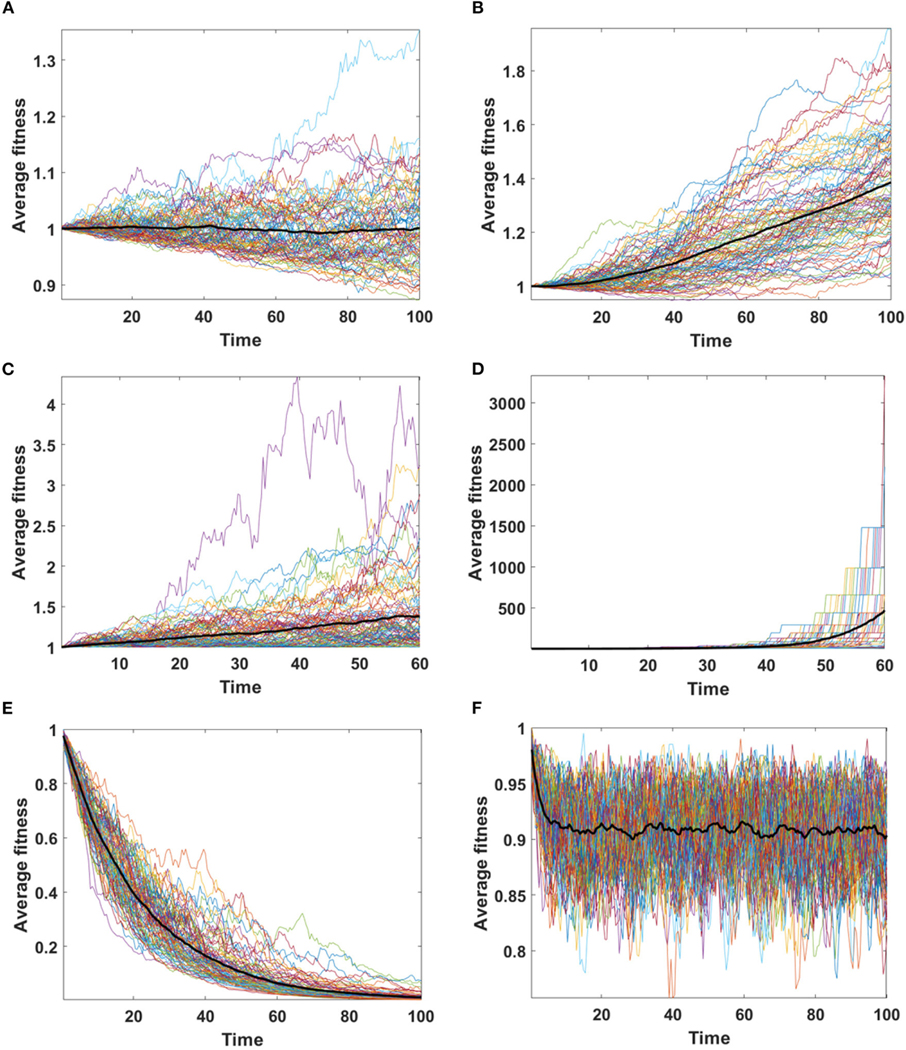
Average fitness of cells as a function of time. Bold lines represent the mean of average fitnesses from 100 simulations. (**A,B**) Results for 100 simulations on N=100 cells in equilibrium (sp=dq) with parameters: s=0.1, d=0.01, μ=0.1, p=0.0909. (**A**) Model A; (**B**) Model B. (**C,D**) Results for 100 simulations on N=100 cells under selection (sp≫dq) with parameters: s=0.5, d=0, μ=0.1, p=0.1. (**C**) Model A; (**D**) Model B. Please note that the time range in plots C and D is 0 to 60, since some of the trajectories grow too fast to be accomodated by computer memory limits. (**E, F**) Results for 100 simulations on N=100 cells under negative selection (sp<dq) with parameters: s=0, d=0.5, μ=0.1, p=0.1. (**E**) Model A; (**F**) Model B.

Assume that the impact of driver and passenger mutations is in equilibrium - the less frequent appearance of drivers is balanced by higher impact *s* on the fitness of given clone. Based on Equation (6) no systematic trend in fitness is expected, as confirmed for Model A simulations (Figure 8A). For Model B, despite accumulating passenger mutation, the average fitness slightly increases, due to the drift process favoring fixation of clones with higher fitness (Figure 8B). The dynamics of fitness increase in the Model B is also different than in the Model A.

**FIGURE 9 |:**
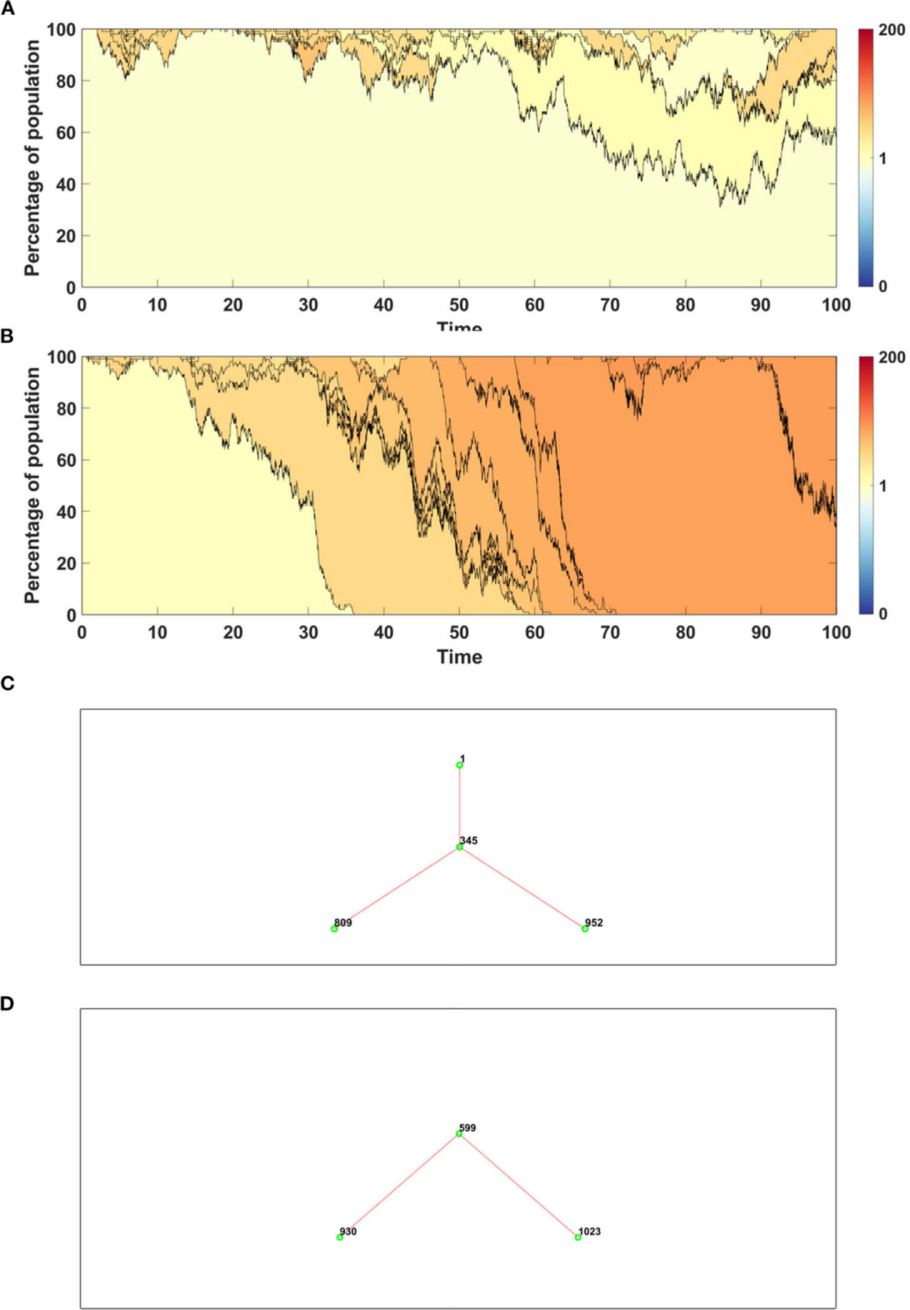
Time succession patterns. Clones started with driver mutations are colored according to average fitness of given clone. Results of one simulation on N=100 cells with parameters: s=0.1, d=0.01, μ=0.1, p=0.0909. (**A**) Model A; (**B**) Model B. Genealogy of clones. Results of one simulation on N=100 cells with parameters: s=0.1, d=0.01, μ=0.1, p=0.0909. (**C**) Model A–genealogy of clones started from driver mutation, alive at t=100 (out of 992 clones emerged through the time of simulation). (**D**) Model B–genealogy of clones started from driver mutation, alive at t=100 (out of 1041 clones emerged through the time of simulation).

The time succession plots (Figures 9A,B) are kept in the same convention as in previous example, but for clarity shown are only clones originating from driver mutation. The fitness of such clone, represented by corresponding shade of color as shown on scale bar next to the figures, is calculated as an average across all passenger clones sharing the same driver mutation.

Results obtained with the use of Model A have higher number of clones with wide spectrum of fitness (Figure 9A), while in the case of Model B a few clones with higher fitness dominate the population (Figure 9B).

In Figure 9 we compare also genealogies of the clones. Results are shown in the form of ancestor-descendant trees depicting relationship between clones, but without specifying the time at which given clone appeared. The numbers next to the circles represent the order number of a clone. For clarity the graphs are showing only clones starting from a driver, alive at t=100. The total number of clones which appeared during the simulation is equal to 992 in the case of Model A (Figure 9C) and 1,041 in the case of Model B (Figure 9D). Driver mutations are marked by red lines. The topology of genealogies in equilibrium case (sp=dq) is similar for both models.

#### 3.1.3. Asymmetric, sp≠d(1−p), Case of Models A and B

We now consider the case in which the impact of driver mutations is high, while the passengers have no impact on fitness of cells. In such case sp>dq, and the average fitness is expected to rise. Expected behavior is observed in results obtained by both, Model A and B (Figures 8C,D); however the shape of trajectories is different due to a much higher number of events observed in Model B (despite similar number of mutations), which leads to the takeover of the population by clones with higher fitness.

**FIGURE 10 |:**
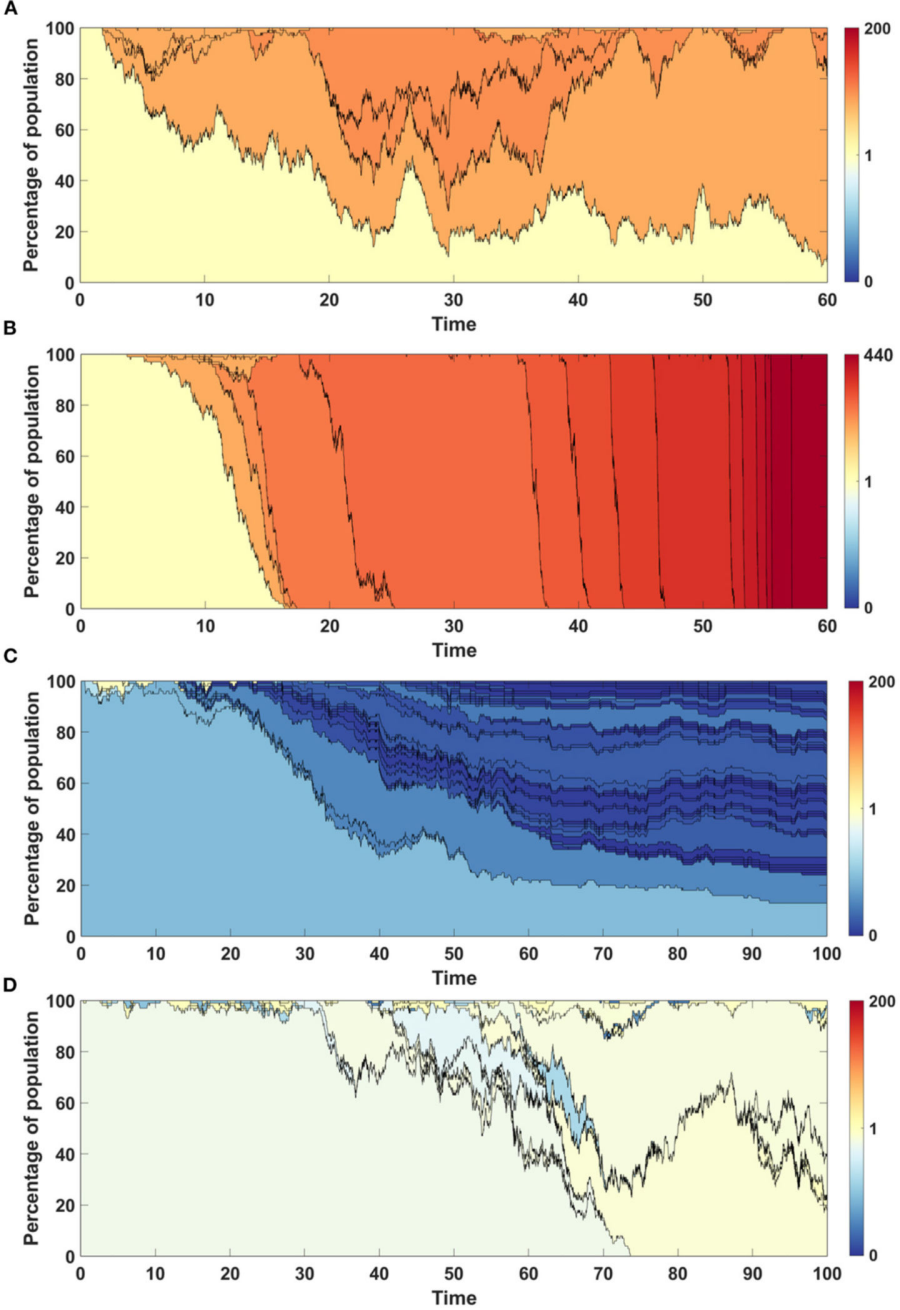
Time succession patterns. Clones started with driver mutations are colored according to average fitness of given clone. (**A,B**) Results of one simulation on N=100 cells with parameters: s=0.5, d=0, μ=0.1, p=0.1 (sp>dq). (**A**) Model A; (**B**) Model B. (**C,D**) Results of one simulation on N=100 cells with parameters: s=0, d=0.5, μ=0.1, p=0.1 (sp<dq). (**A**) Model A; (**B**) Model B.

The time succession patterns and the number of living clones at the end of simulation also vary substantially between both Models (Figures 10A,B and Supplementary Figures S1A,B).

In the last scenario, we consider situation with no impact of driver mutations, but high impact of passenger mutations (sp≪dq). Results demonstrate very clearly the qualitative differences between A and B Models that may be overlooked in more balanced examples.

Based on Equation (6) we expect falling values of average fitness as seen in Model A result (Figure 8E). The behavior of average fitness in Model B simulations does not fit well into these expectations. After initial period of decreasing, the average fitnesses are balancing around certain level, not dropping to zero (Figure 8F).

Time succession plots also differ between the results of simulations of models A and B. In the case of Model A, large number of clones with low fitnesses emerge (Figure 10C), while in Model B, these are continually removed from the population and replaced by clones with fitness close to 1 (Figure 10D). Note that in both figures, only clones starting from driver mutations are presented, which indicates that in the case of Model A, driver mutations arise on the background of the large number of passenger mutations and are not able to overcome this effect, while in the case of Model B, clones with passenger mutations are not fixed in the population, but are replaced by clones with higher number of drivers.

The difference between both Models is noticeable also in clone genealogies (Supplementary Figures S1C,D), with Model A having much more clones alive at the end of simulation. The overall number of events is higher in case of Model B, but the percentage of mutation events is much higher in case of Model A.

#### 3.1.4. Neutrality Tests

In the following section we present the outcome of neutrality testing (see earlier on) of the simulation results obtained using Models A and B. In all cases tests were performed on samples including 1,000 simulation results. Top panels show the comparison between the number of alleles (k) observed in simulation results and the number of alleles which is theoretically expected, while bottom panel presents number of singletons expected and observed in simulation results. In the latter case narrower red bars representing simulated spectra of singletons are placed “in front” of expected spectra (blue bars).

One-sample Kolmogorov-Smirnov tests were performed to examine the hypothesis that the empirical distribution of allele count fits the theoretical one (Models A and B). To examine the hypothesis that simulated singleton counts fitted the distribution simulated under neutrality, we performed two-sample two-sided Wilcoxon tests.

**FIGURE 11 |:**
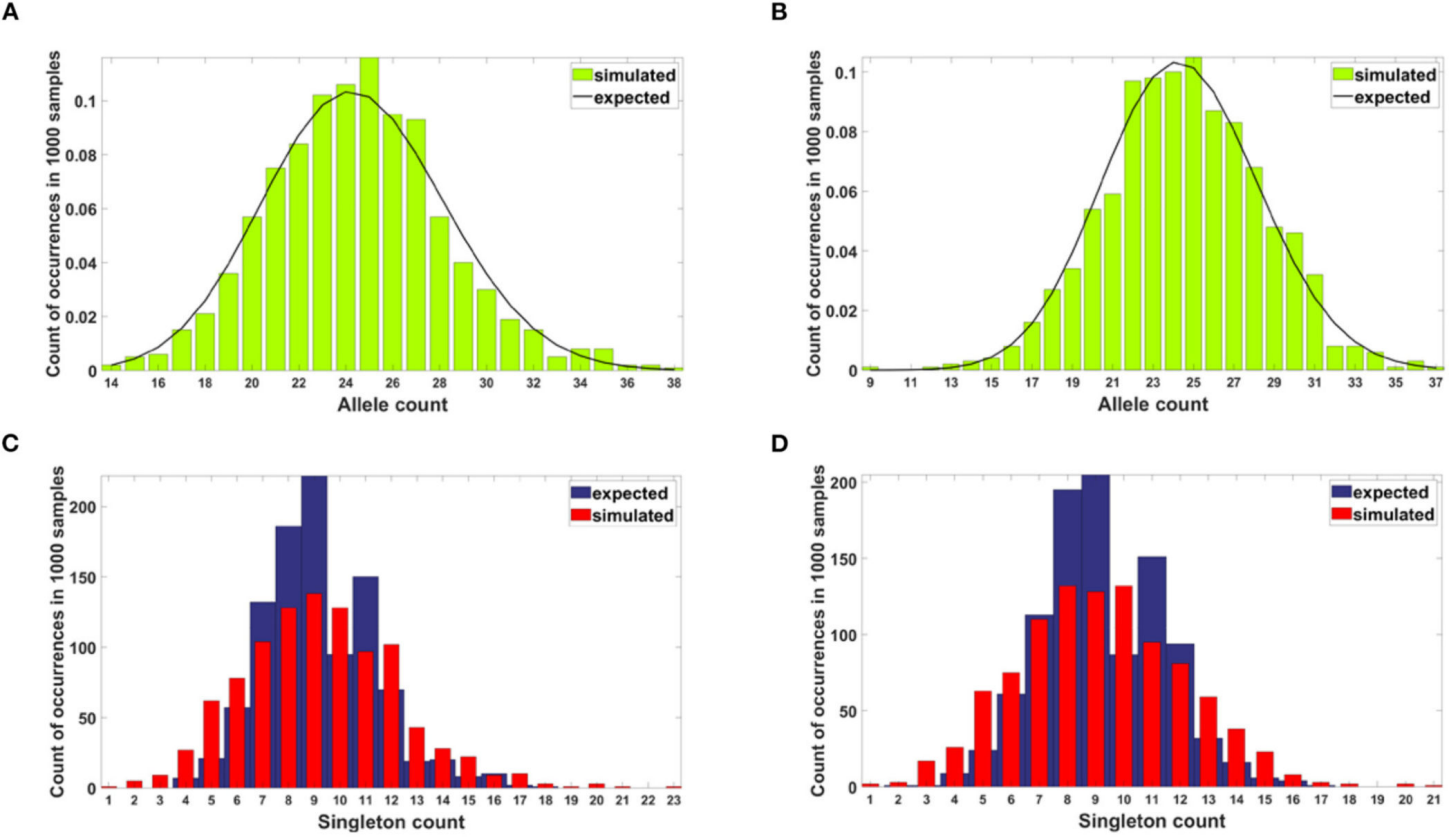
Results of neutrality testing for allele counts (top panels) and singleton number (bottom panels) calculated for simulations with parameters: s=0, d=0, μ=0.1, p=0.5. (**A**) Model A. One-sample Kolmogorov-Smirnov test does not reject the null hypothesis that the empirical distribution of allele count fits the expected one at the 5% significance level (p=0.62). (**B**) Model B. One-sample Kolmogorov-Smirnov test does not reject the null hypothesis that the empirical distribution of allele count fits the expected one at the 5% significance level (p≈1). (**C**) Model A. Two-sample two-sided Wilcoxon test does not reject the null hypothesis that simulated and expected singleton counts come from distributions with equal medians at 5% significance level (p=0.48). (**D**) Model B. Two-sample two-sided Wilcoxon test does not reject the null hypothesis that simulated and expected singleton counts come from distributions with equal medians at 5% significance level (p=0.83).

Figure 11 shows the neutrality testing outcomes for the case with no impact of driver or passenger mutations (s=d=0). Note the difference between the following example and the case described in Section 3.1.1: mutation process is present here, but mutations do not affect cell fitness.

In the case of both Models, the simulated allele counts adhere to expected values (Figures 11A,B). The empirical singleton counts in both cases (Figures 11C,D) slightly differ from expectations in shape but not in central tendency.

We performed the same type of analysis for the remaining cases. Results are presented in Supplementary Figures S2–S4.

### 3.2. Comparison of Simulated SFS to Data

#### Obtained From Breast Cancers

Semi-logarithmic cumulative tails of variant allele frequencies (VAF) from the breast cancer samples G30 and G31 from our collection have been compared to the semi-logarithmic cumulative tails of SFS generated from model A and model B simulations.

Here we present the results for raw and resampled data (details regarding reducing coverage bias procedure are described in Section 2.6.2).

Simulated datasets trace the sensitivity to varying

**FIGURE 12 |:**
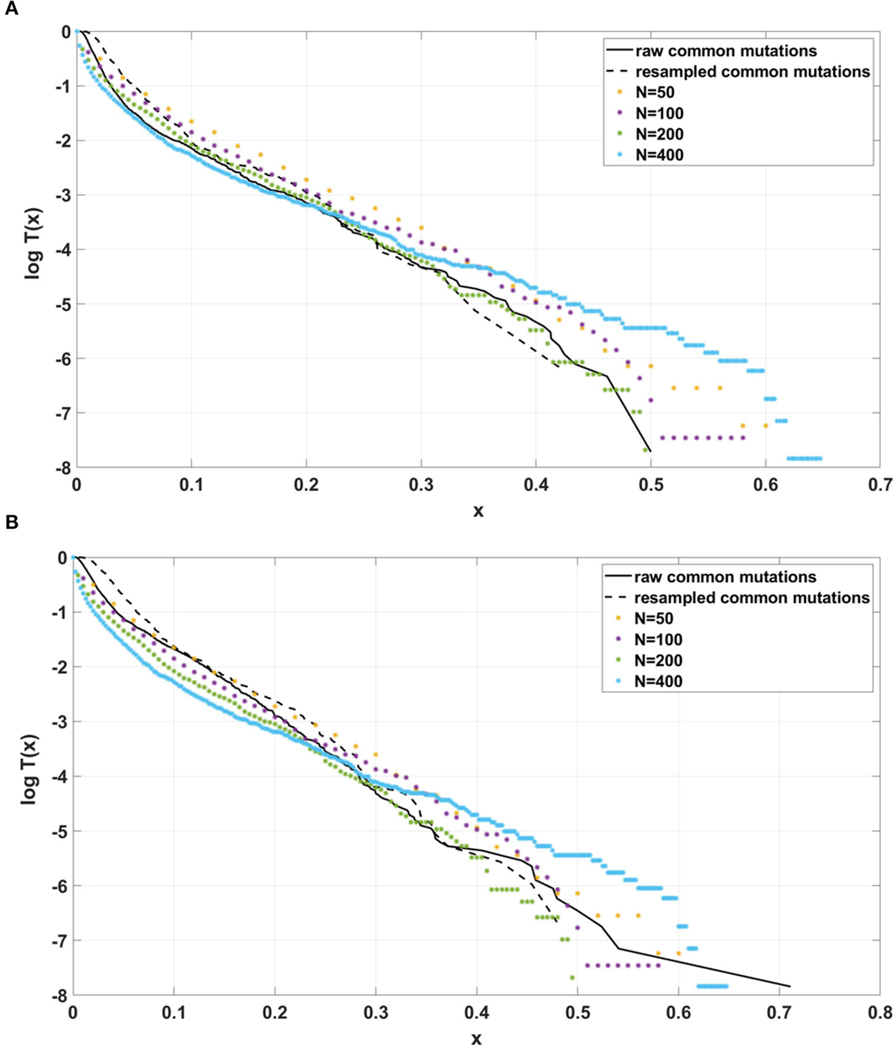
Comparison of semi-logarithmic cumulative tails of the SFS obtained for experimental data from (**A**) patient G30 and (**B**) patient G31 with four sets of simulations with parameters: s=0.5, d=0.0001, p=0.01, L=Nμ=6, and variable population size N.

**FIGURE 13 |:**
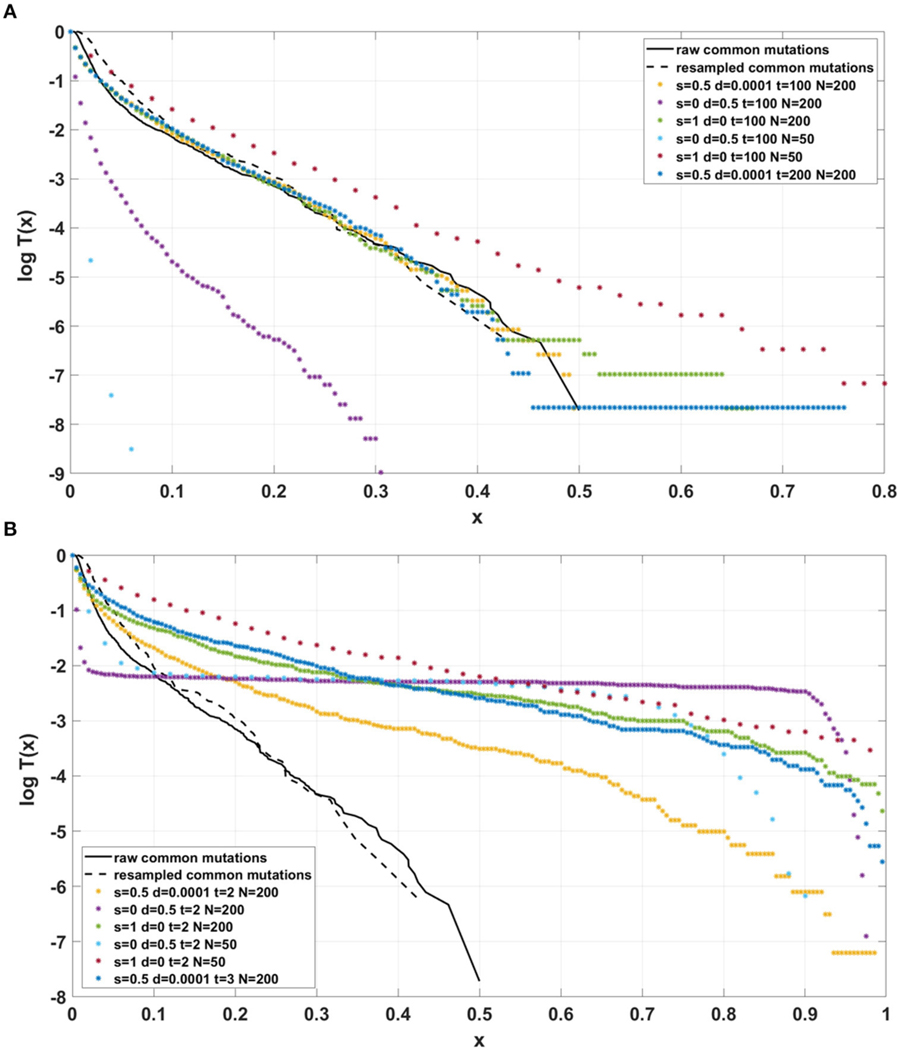
Comparison of semi-logarithmic cumulative tails of the SFS obtained for experimental data from patient G30 with average from 100 simulations with parameters L=Nμ=6 and p=0.01. (**A**) Model A; (**B**) Model B.

**FIGURE 14 |:**
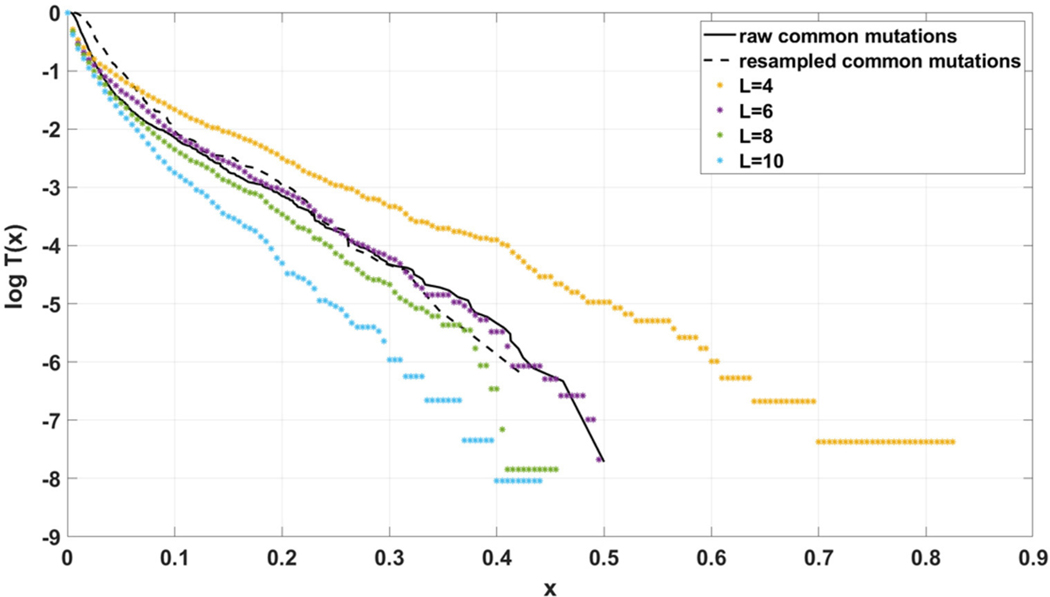
Comparison of semi-logarithmic cumulative tails of the SFS obtained for experimental data from patient G30 to simulations with Model A with varying μ. Average from 100 simulations on 200 cells at time t=100. s=0.5, d=0.0001, p=0.01, N=200.

- the number of individuals N, with a concurrent change in μ, so the product of both remains constant (Figure 12),
- the s and d coefficients (Figures 13A,B and Supplementary Figure S5),
- the simulation time (Figures 13A,B and Supplementary Figure S5), and
- the mutation rate μ, with population size N kept constant (Figure 14).

Additionally, for both Models we explored following parameter values: (i) d=0, d=0.001, d=0.1 (ii) s=0, s=0.5, s=1; (iii) L=1, L=5, L=10 (in all possible combinations, with p=0.01 and N=200, see Supplementary Figures S10–S12). In all cases simulation time was equal t=100.

The site frequency spectra are calculated based on observed allele frequency at the end of simulation (t=100 in all cases) and presented as semi-logarithmic cumulative tails.

For the two patients G30 and G31, semi-logarithmic cumulative tails of the SFS obtained from Model A fit the experimental data well (Figures 12A,B, 13A), while results obtained with the use of model B do not fit the tails of VAF from breast cancer sample G30 (Figure 13B) or G31 (Supplementary Figure S5).

## 4. DISCUSSION

The analysis of the two models we discuss in the current paper is relevant for two topics widely discussed in population genetics. One of them, of current interest, is in what sense the evolution in cancers is “Darwinian.” The other, with much more profound roots, concerns the interaction of mutation, drift and selection in asexual populations.

In our Model A the drift and selection component does not increase fitness as demonstrated by Equation (4). Expected fitness behaves precisely as predicted by the mutation balance, increases with the drivers prevailing (sp>dq), decreases with passengers prevailing (sp<dq), and remains constant at mutational equilibrium (sp=dq).

Model B, patterned after the model introduced in Bobrowski et al. (2021), behaves in a more complex manner. The drift and selection component increases expected fitness as demonstrated by Equation (5). Expected fitness cannot be predicted by the mutation balance only, although it increases with the drivers prevailing (sp>dq). However, with passengers prevailing (sp<dq), the fitness may decrease or increase depending on how much smaller sp is than d(1−p). Fitness generally increases at mutational equilibrium (sp=dq). These effects seem consistent with the so-called drift barrier, which prevents the deleterious passenger mutations from dominating fitness change too easily.

One of the fundamental problems in understanding the evolution of cancerous tumors is the pattern of selection present there. In blood tumors, which evolve mostly in the bone marrow, the disease is confined to a restricted environment, although cellularity of the bone marrow tends to be increased (see the analyses in Dinh et al., 2021). Moran model with selection was shown to lead to predictions consistent with clinical findings in evolution of myeloid dysplastic syndrome from severe congenital neutropenia (Wojdyla et al., 2019; Dinh et al., 2020a). In solid tumors, the growth is more expansive, although a range of growth patterns are present. Ling et al. (2015) presented an analysis of a cross-section of human liver cancer, sampled genomically in around 300 locations, which seems to demonstrate lack of departure from the neutral mutation Infinite Sites Model, based on the analysis of SFS using the Durett’s approximate formula (Durrett, 2013). More recently, McDonald et al. (2018) argued that available bulk sequencing data do not necessarily support a model of neutral tumor evolution, based on a birth-and-death process model. This model was recently analyzed mathematically by Tung and Durrett (2021), who state it may be used as another test of “Darwinian” selection.

In the realm of neutral theory, a large number of models were developed, from which analytical or at least computational expressions for the expectation of SFS can be derived. The classical model of Griffiths and Tavaré (1998) includes, among other, an expression for the SFS under Wright-Fisher model with arbitrarily varying population. This expression was rearranged to make it computable for large samples by Polanski and Kimmel (2003). In the case of exponential population growth, Durrett (2013) provided an approximate large sample and large population expression, which leads to the conclusion that, in this case, the SFS cumulative tail in the log-log scale should be approximated by a straight line with coefficient −1. Working directly with a birth-death process with binomial sampling, Lambert and Stadler (2013) developed an SFS expression, which is easily computable and surprisingly leads to curves very similar to Durrett’s approximation of Griffths-Tavaré’s SFS (see a comparison in Dinh et al., 2020b).

Against this background, we now discuss our tug-of-war type Models A and B. Let us note that Dinh et al. (2020b) developed a model of neutral evolution with selective sweeps, which generates humps overlapping the neutral Griffths-Tavaré’s spectrum. This model seems appropriate for tumors with distinctive genomic clones, such as lung cancer, displaying “punctuated” evolution (Davis et al., 2017). However, in our breast cancer spectra (e.g., Figures 12A,B), we do not observe “humps,” although it was suggested in Gao et al. (2016) that the evolution of aneuploidy in breast cancer proceeds in a punctuated manner. In addition, it is known that under neutral Wright-Fisher (or Moran) model as, e.g., recently studied by Gunnarsson et al. (2021), the SFS tails are approximately $x^{-2}$ if the population is growing exponentially, and $x^{-1}$ if it is constant. Models A and B exhibit power-law tails if s=d=0, i.e., under “strict” neutrality. The $x^{-2}$ SFS tail implies the $x^{-1}$ cumulative tail T(x), leading to straight line with −1 slope in log-log coordinates. Tung and Durrett (2021) paper also implies a power law, albeit a different one. Power laws in general predict a decreasing straight-line cumulative SFS tail when plotted in the log-log scale. In contrast, in the breast cancer data-based spectra, the cumulative SFS tails seem slightly convex in semi-logarithmic scale, but concave in log-log coordinates (Supplementary Figures S6–S9), this latter making them inconsistent with power laws.

If the SFS of the breast cancer specimens G30 and G31 are compared to predictions of Models A and B, it becomes clear that it is Model A, which is fitting at least approximately, and not Model B. To see this, compare Figure 13A to Figure 13B. The striking qualitative difference between SFS cumulative tails T(x) generated by Model A and by Model B is preserved for a wide range of parameter values. Specifically, Figure 13 and Supplementary Figures S10–S12 demonstrate this for a range of parameters s, d, L and p. In addition, extending time beyond t=100 does not alter the SFS markedly. Figures 12A,B demonstrate that increasing N seems to change only slightly the simulated cumulative SFS tail for given s and d, provided parameter L=Nμ stays constant, except for the “deep tail” below the $T(x)=10^{-4}$ mark. On the contrary, in case when population size N is constant and the varied parameter is mutation rate μ (impacting L parameter value), the corresponding SFS cumulative tail trajectories vary more (Figure 14).

How does the Model A fit to cancer data relate to biological parameters? Let us note that for L=Nμ=6, which provides feasible fits to the empirical SFS tails in Figure 12, if N=400 is accepted, we obtain mutation rate μ=0.0175 [time unit^−1^], which is not very different from the expected rate per exome (ca. 1% of genome) per cell division. This corresponds to the time scale of single model time unit per division. This scaling is appropriate if we apply the model to a population of several hundred cells. This is equivalent to the typical coverage value of a bulk sequencing sample (for details, cf. Dinh et al., 2020b). If the effective population size is larger, the time scale has to be different, which will affect the transients of the model. Another context in which these estimates are realistic, is under assumption that the clonal structure of tumors is due to a small count (10^2^ – 10^4^) of cancer stem cells, having indefinite proliferative potential, with the rest of tumor cells capable of only limited division count.

**FIGURE 15 |:**
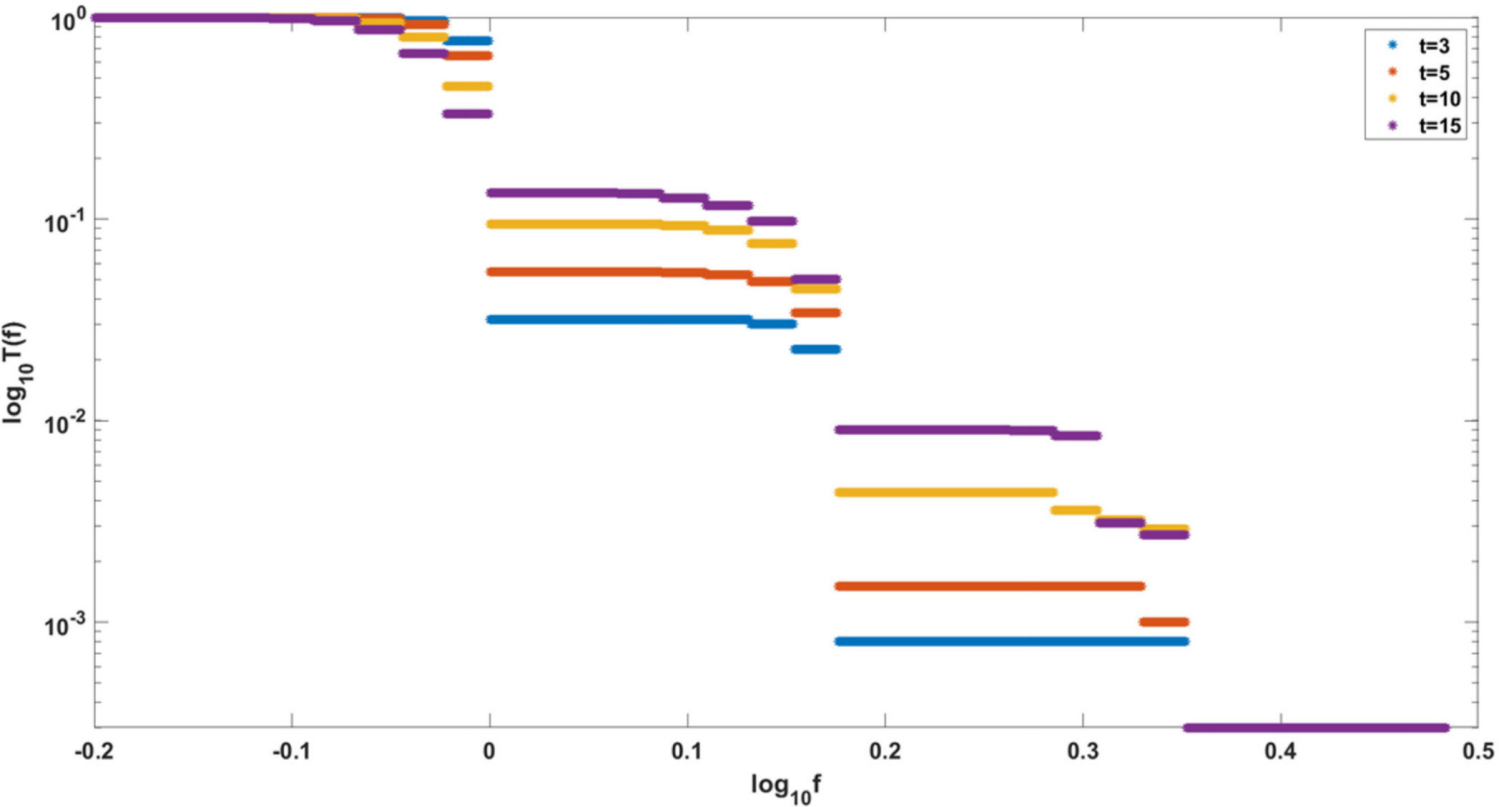
Evolution of clones with increasing fitness, according to Model A. Depicted are logarithmic cumulative distribution tails $\log_{10}T(f)$ of logarithmic fitness $\log_{10}f$, for a series of times t=3,5,10,15. Parameter values, s=0.5, d=0.05, p=0.1, L=Nμ=10.

Finally, let us note that it sometimes makes sense to consider cancer growth in the framework of constant-population models. Our models correspond to the situation in which a constant population of N “healthy” stem cells is gradually replaced by a growing clone of transformed cells with increasing fitness. This is exemplified in the simulation in Figure 15, which shows the gradual rise of a clone with fitness up to several times the fitness of initial “healthy” cells. This is very similar to evolution of relapsing leukemic clones as in Dinh et al. (2021).

We acknowledge that our approach is related to the past theoretical work in the field of evolutionary genetics, such as Peck (1994), Johnson and Barton (2002), Bachtrog and Gordo (2004), Good and Desai (2014, 2015), and Rouzine et al. (2003). These papers concern the interplay among mutation, drift and selection, in absence of recombination (asexual reproduction), where epistasis plays a major role. Most of these papers concern the role of the drift barrier and effects such as Muller’s ratchet. There exist similarities and differences between these models and ours, analysis of which requires much more research than possible here.

In addition in Supplementary Material Part 2, we illustrated comparisons of the model-generated SFS cumulative tails (Models A and B, model of Tung and Durrett (2021), and the multiclone model of Dinh et al. (2020b), to SFS obtained from 4 TCGA cancers, breast, prostate, skin melanoma, and ovarian. Among breast cancers, we found a number that fit Model A or model B, while prostate cancers seem to not fit Models A or B. Melanoma and ovarian cancers SFS frequently conform to the multihump (Dinh et al., 2020b) model. As for Tung and Durrett (2021), this model produces tails similar to a case of Model B. These comparisons illustrate a wide range of patterns of SFS in different cancer types.

As noted in Results and Supplementary Materials, neutrality tests based on the distribution of singleton counts and Ewens Sampling Formula indicate deviations from null hypotheses for both Model A and model B, except for the “truly neutral” case of s=d=0. A related question is behavior of Models A and B under population growth, such as in a branching or birth-death process model.

To conclude, two different Moran-type models of the Tug-of-War process are based on underlying drift and selection mechanism which either preserves expected fitness (Model A) or is biased toward fitness increase (Model B). Therefore, fitness change in Model A depends on the mutational balance only (see Section 2.2.3). Based on simulation results, Model A and Model B are leading to SFS with the qualitative difference persisting over a range of parameters and times. Model A seems to better fit the HER2+ breast tumor data. Model A is consistent with the fitter cells reaching division (“dying”) faster and being replaced preferentially by fitter cells. This evolution mode is also “Darwinian,” but leading to different SFSs.

## Supplementary Material

Data Sheet 1.pdf

## Figures and Tables

**Table 1.** Descriptive statitics of reads from DNA sequencing of genomes of the 2 breast cancer used as an illustration.

SampleID	raw total sequences	filtered sequences	filtered sequences [%]	sequences	reads mapped	reads mapped and paired	reads unmapped	reads properly paired	reads paired	reads MQ0	non-primary alignments	total length	bases mapped	bases mapped (cigar)	mismatches	error rate	average quality	insert size median	insert size average	insert size standard deviation	inward oriented pairs	outward oriented pairs	pairs with other orientation	pairs on different chromosomes	percentage of properly paired reads (%)
G30_C	107474166	24048910	22.3764564965315	83425256	83289285	83233642	135971	76532560	83425256	3333413	21483890	12429700477	12411110876	11011895244	48789146	0.004430586	28.4	166	169.9	59.1	30073176	8430757	235545	2877343	91.7
G30_L1	103818284	25135709	24.2112545416374	78682575	78567070	78524876	115505	74932230	78682575	2957434	11450725	11726432892	11710529621	10755430225	43584266	0.004052303	29.3	172	175.1	53	29778856	7817748	123559	1542275	95.2
G30_P1	118937268	30440295	25.5935717306034	88496973	88397893	88379716	99080	87521806	88496973	2226389	1325724	13216412290	13202330029	13032563995	38715779	0.002970696	32.5	212	216.2	53.7	40825643	2994772	45078	324365	98.9
G31_C	109506560	29801374	27.2142362978072	79705186	79638653	79614338	66533	76006316	79705186	2681243	7349701	11890693003	11881999787	11266252670	32815041	0.002912685	31.8	188	193.4	58.6	32429386	5779868	191240	1406675	95.4
G31_L1	100518046	26734630	26.5968461026391	73783416	73715495	73690438	67921	69101166	73783416	2295598	9807154	11008840326	10999976250	10367481277	32414713	0.003126576	31.5	198	203.4	65	30444228	4301517	201987	1897487	93.7
G31_P1	104211788	29667651	28.4686133587882	74544137	74485369	74464096	58768	71592980	74544137	2191887	5616199	11122462545	11114771921	10613831783	30572697	0.002880458	32	194	199	59.8	31046638	4927073	182959	1075378	96

Abbreviations: P1 - primary tunor, L1 - lymph-node metastasis, C - common part

## Data Availability

The original contributions presented in the study are publicly available. This data can be found here: https://ega-archive.org/with accession number: EGAC00001002785. Any queries should be directed to the corresponding author.
