# Supplementary material for: Modes of Selection in Tumors as Reflected by Two Mathematical Models and Site Frequency Spectra
Source: Front Ecol Evol. Author manuscript; Available in PMC 2023 Jun 16. (PMC10275603; doi:10.3389/fevo.2022.889438)
Supplement: Data Sheet 1.pdf [file NIHMS1895874-supplement-Data_Sheet_1_pdf.pdf]

Supplemental Material

1 SUPPLEMENTAL TABLES AND FIGURES

1.1 Genealogies of the clones – cases with selection

1.1.1 Mutations increasing expected fitness ($sp > dq$)

Genealogies of clones shown in Figure S1 A and B indicate that strong selection present in both simulated populations results in the small number of clones living to the end of simulation. Additionally, as may be seen in Figure S1B, these clones likely descend from one dominating driver ancestry line.

1.1.2 Mutations decreasing expected fitness ($sp < dq$)

In the case of Model A, the large number of living clones started from a driver are present at the end of simulation (Figure S1C). Many of them descend directly from the initial clone. Each of clones includes a small number of cells. In contrast, the result obtained with use of Model B (Figure S1D) indicate less clones present at the end of simulation, with initial clone no longer present in the population. Mutations not connected to any ancestor (clones 521 and 594 in Figure S1D descend from the initial clone, with possible earlier passenger mutations.

1.2 Neutrality testing

1.2.1 Mutations preserving expected fitness ($sp = dq$)

In the population where impacts of driver and passenger mutations are in equilibrium, the allele count spectrum have different shape than expected under neutrality in case of model B, (Figure S2B), while in case of model A observed allele count fits better to the expectations (Figure S2A). The count of singletons calculated based on simulations with use of both Models correspond well to the expected one (Figure S2C and D).

1.2.2 Mutations increasing expected fitness ($sp > dq$)

In the case of higher driver mutations impact, results obtained with use of model B (Figure S3 B and D), especially allele count (Figure S3B), differ substantially from expected ones. Model A generates singleton count spectrum corresponding well to the expected distribution (Figure S3C), but observed allele count spectrum deviates significantly from the theoretical curve (Figure S3A).

1.2.3 Mutations decreasing expected fitness ($sp < dq$)

In the last example, both models yield allele count spectra with significant deviation from neutrality (Figure S4 A and B). In this case, the effect is stronger for spectrum generated by model A (Figure S4A). However, for this Model We observe similar number of singletons as expected for given allele count S4C, which is not the case for Model B, where simulated and expected singleton counts differ significantly (Figure S4D).

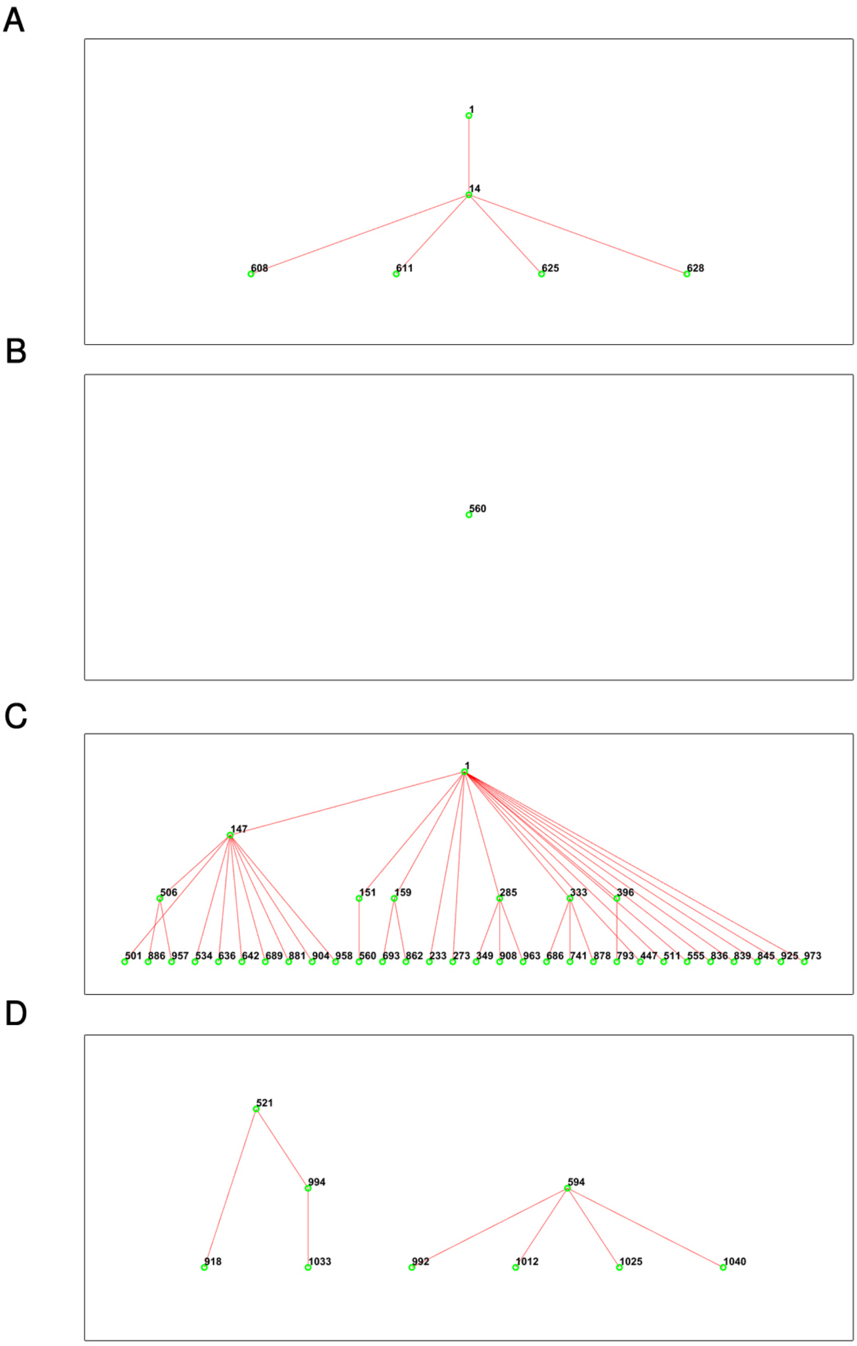

Figure S1. Genealogy of clones. (A-B) Results of one simulation of $N = 100$ cells with parameters: $s = 0.5, d = 0, \mu = 0.1, p = 0.1$. **(A)** Model A – genealogy of clones started from driver mutation, alive at $t = 60$ (out of 630 clones that emerged over the time of simulation). **(B)** Model B – genealogy of clones started from driver mutation, alive at $t = 60$ out of 588 clones that emerged over the time of simulation). (C-D) Results of one simulation of $N = 100$ cells with parameters: $s = 0, d = 0.5, \mu = 0.1, p = 0.1$. **(C)** Model A – genealogy of clones started from driver mutation, alive at $t = 100$ (out of 983 clones emerged through the time of simulation). **(D)** Model B – genealogy of clones started from driver mutation, alive at $t = 100$ (out of 1045 clones that emerged over the time of simulation).

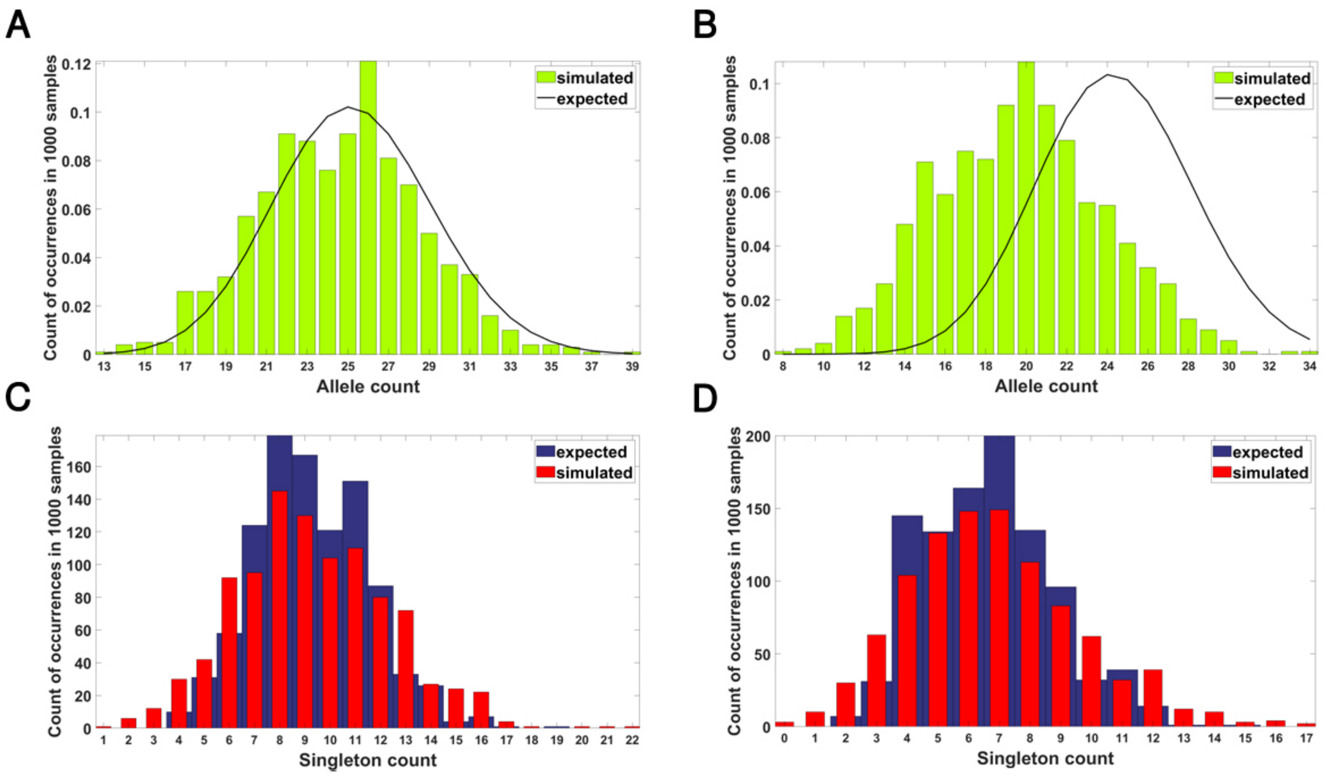

Figure S2. Results of neutrality testing for allele counts (top panels) and singleton number (bottom panels) calculated for simulations with parameters: $s = 0.1, d = 0.01, \mu = 0.1, p = 0.0909$. **(A)** Model A. One-sample Kolmogorov–Smirnov test rejects the null hypothesis that the empirical distribution of allele count fits the theoretical one at the 5% significance level ($p < 0.0001$).
(B) Model B. One-sample Kolmogorov–Smirnov test rejects the null hypothesis that the empirical distribution of allele count fits the theoretical one at the 5% significance level ($p < 0.0001$). **(C)** Model A. Two-sample two-sided Wilcoxon test does not reject the null hypothesis that simulated and expected singleton counts come from distributions with equal medians at 5% significance level ($p = 0.76$).
(D) Model B. Two-sample two-sided Wilcoxon test does not reject the null hypothesis that simulated and expected singleton counts come from distributions with equal medians at 5% significance level ($p = 0.82$).

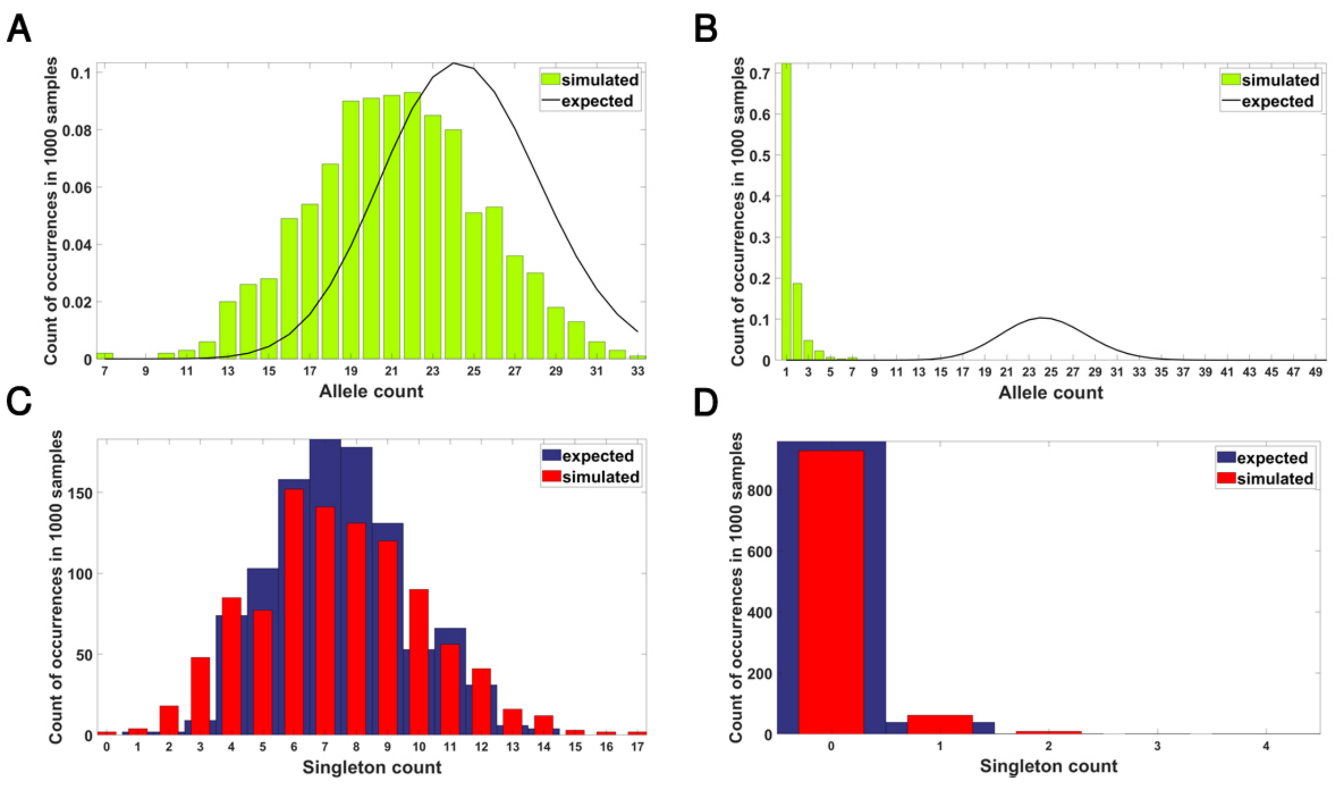

Figure S3. Results of neutrality testing for allele counts (top panels) and singleton number (bottom panels) calculated for simulations with parameters: $s = 0.5, d = 0, \mu = 0.1, p = 0.1$. **(A)** Model A. One-sample Kolmogorov–Smirnov test rejects the null hypothesis that the empirical distribution of allele count fits the theoretical one at the 5% significance level ($p < 0.0001$).
(B) Model B. One-sample Kolmogorov–Smirnov test rejects the null hypothesis that the empirical distribution of allele count fits the theoretical one at the 5% significance level ($p < 0.0001$). **(C)** Model A. Two-sample two-sided Wilcoxon test does not reject the null hypothesis that simulated and expected singleton counts come from distributions with equal medians at 5% significance level ($p = 0.91$).
(D) Model B. Two-sample two-sided Wilcoxon test rejects the null hypothesis that simulated and expected singleton counts come from distributions with equal medians at 5% significance level ($p < 0.0001$).

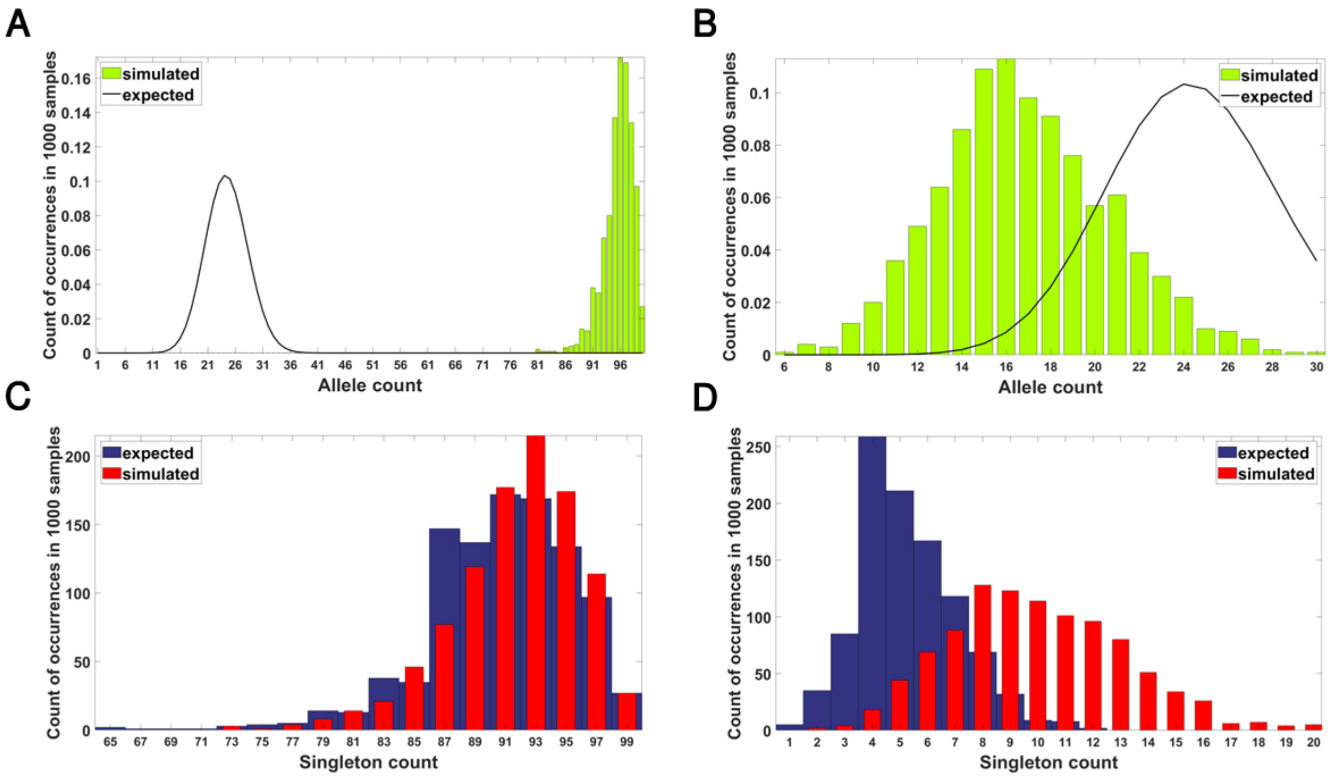

Figure S4. Results of neutrality testing for allele counts (top panels) and singleton number (bottom panels) calculated for simulations with parameters: $s = 0, d = 0.5, \mu = 0.1, p = 0.1$. **(A)** Model A. One-sample Kolmogorov–Smirnov rejects the null hypothesis that the empirical distribution of allele count fits the theoretical one at the 5% significance level ($p < 0.0001$).
(B) Model B. One-sample Kolmogorov–Smirnov test rejects the null hypothesis that the empirical distribution of allele count fits the theoretical one at the 5% significance level ($p < 0.0001$).
(C) Model A. Two-sample two-sided Wilcoxon test does not reject the null hypothesis that simulated and expected singleton counts come from distributions with equal medians at 5% significance level ($p = 0.54$).
(D) Model B. Two-sample two-sided Wilcoxon test rejects the null hypothesis that simulated and expected singleton counts come from distributions with equal medians at 5% significance level ($p < 0.0001$).

1.3 Comparison of simulated cumulative tails of the SFS to experimental data obtained from patient G31

We supplement the comparisons in Figure 13 in the main text, which were performed for experimental data obtained from patient G30, with analogous comparisons for data from patient G31.

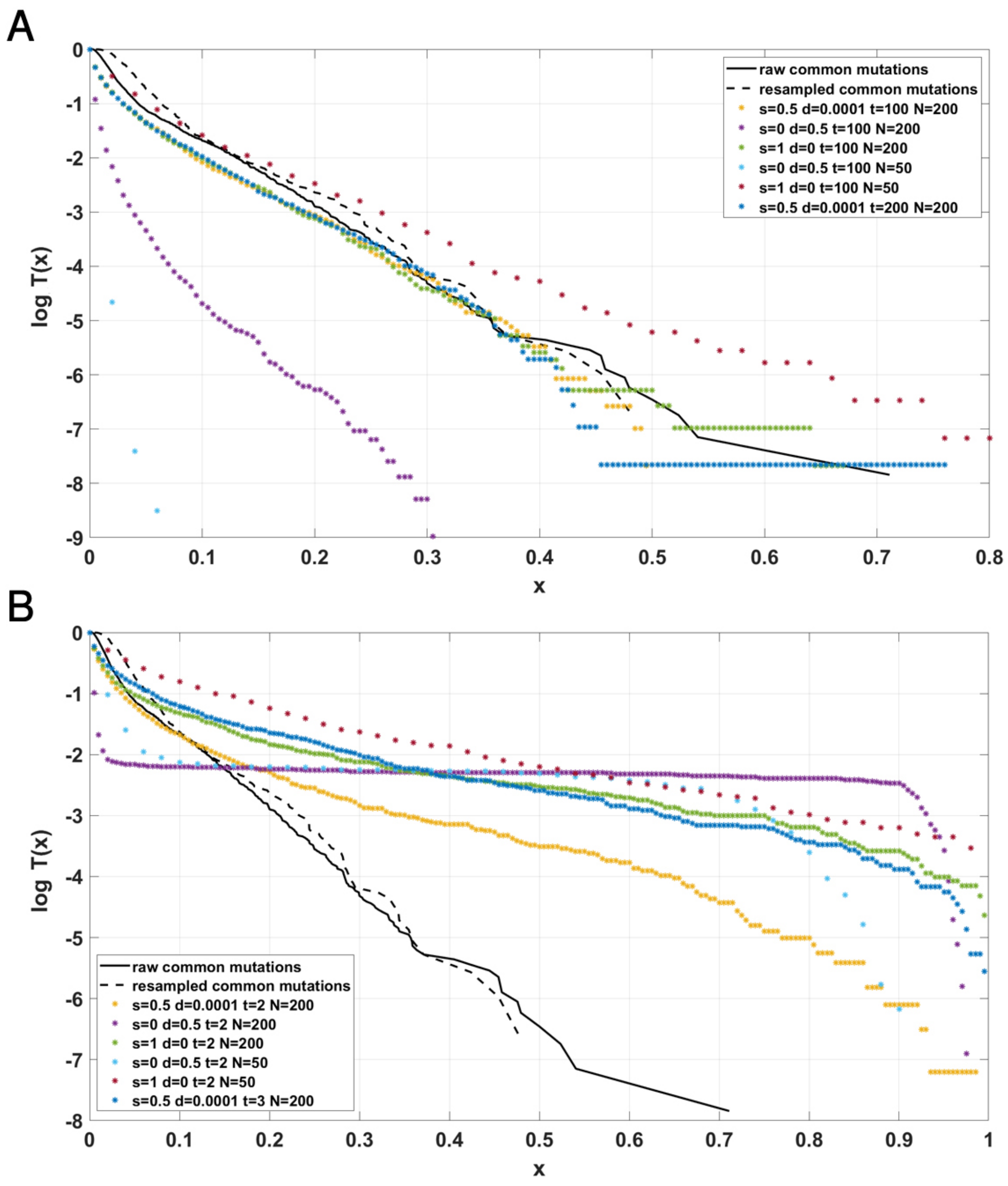

Figure S5. Comparison of semi-logarithmic cumulative tails of the SFS obtained for experimental data from patient G31 with average from 100 simulations with parameters $L = N\mu = 6$ and $p = 0.01$. (A) Model A; (B) Model B.

1.4 Comparison of simulated cumulative tails of the SFS to data obtained from breast cancers – in the log-log scale

We supplement the comparisons in Figures 12, 13 and 14 in the main text and Figure S5, which were depicted in semi-logarithmic coordinates, with analogous comparisons in the log-log scale.

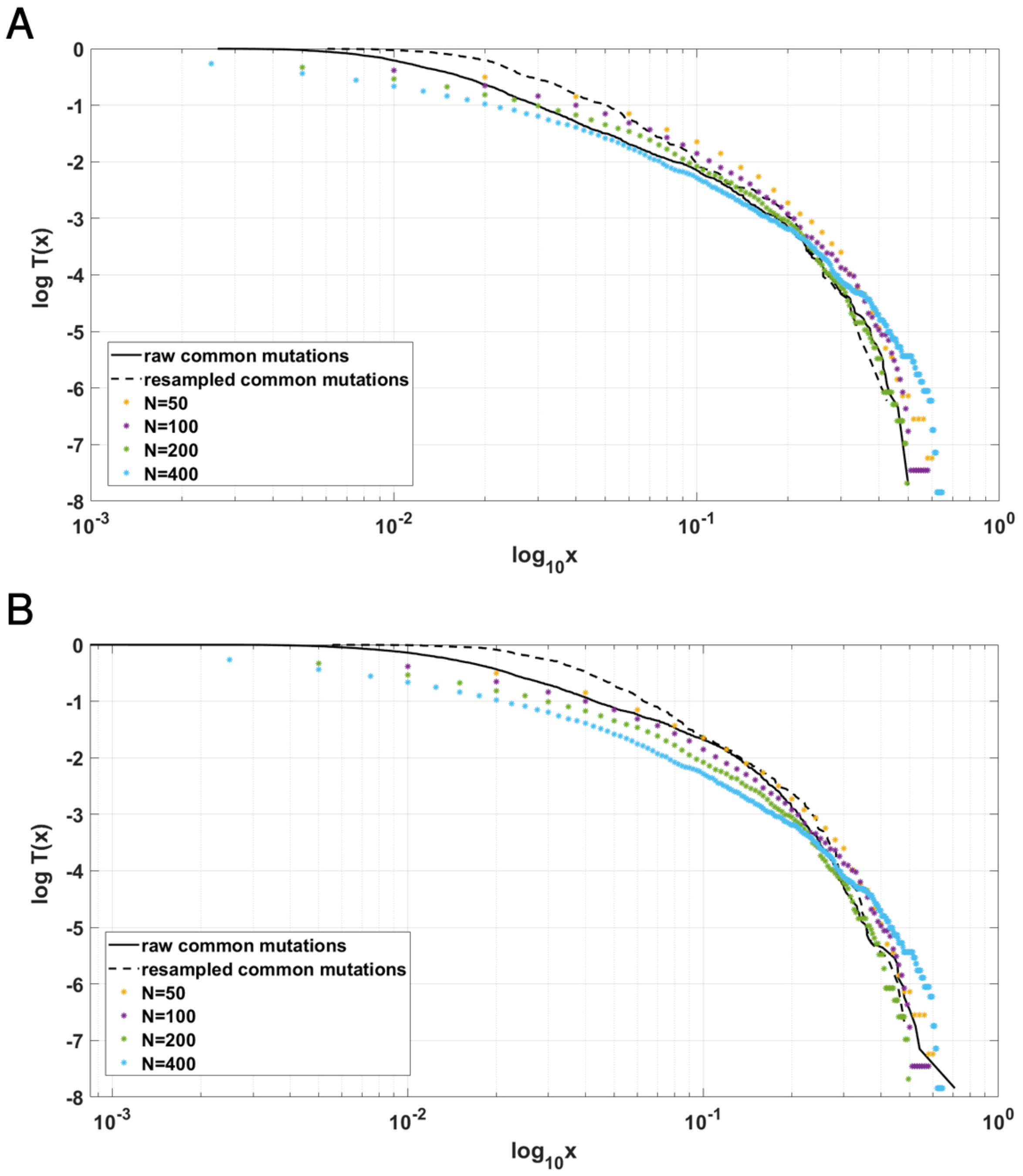

Figure S6. Comparison of log-log cumulative Model A tails of the SFS obtained for experimental data from (A) patient G30 and (B) patient G31, to four sets of simulations with parameters: $s = 0.5$, $d = 0.0001$, $p = 0.01$, $L = N\mu = 6$ and varied population size N.

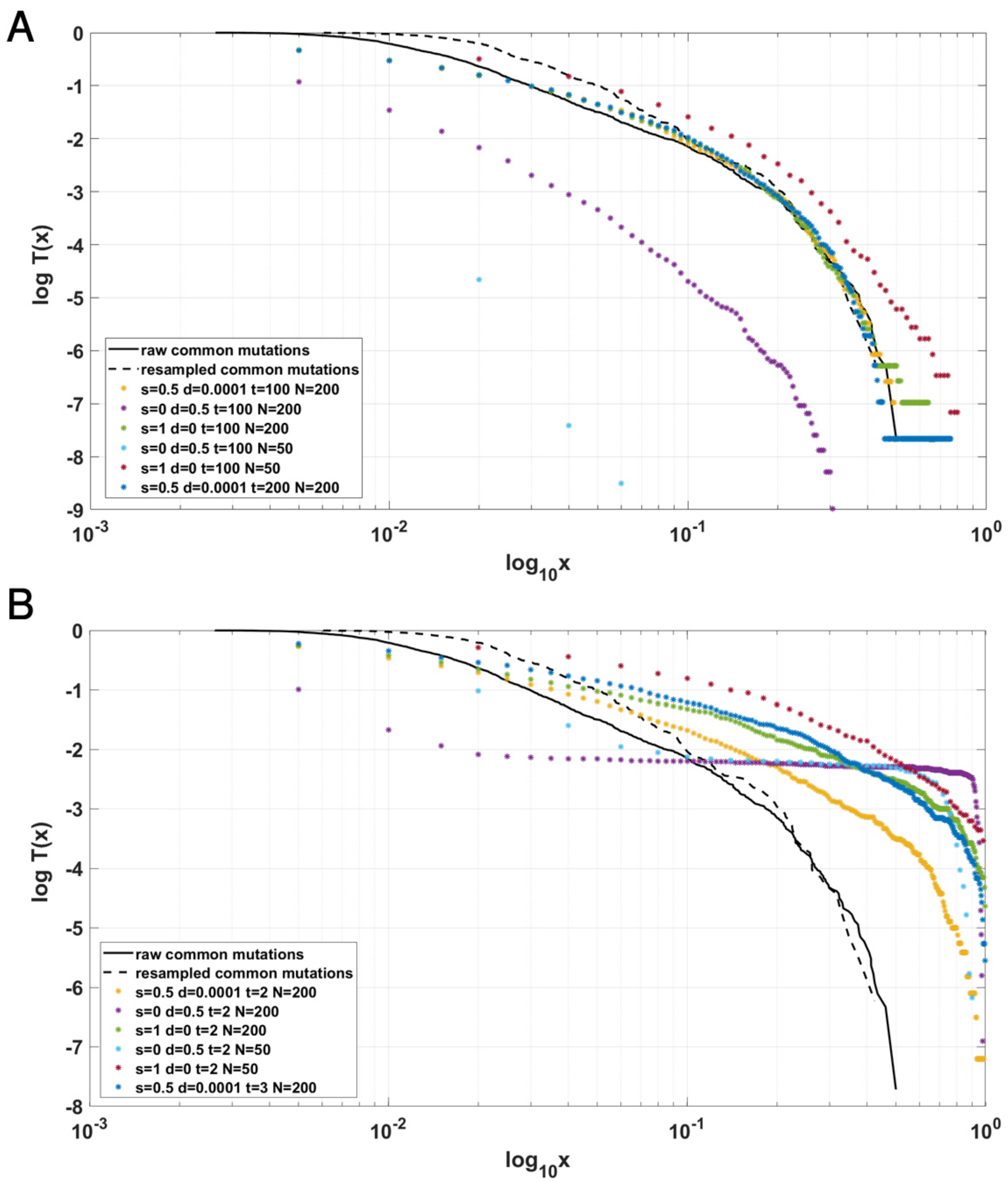

Figure S7. Comparison of log-log cumulative tails of the SFS obtained for experimental data from patient G30, to an average of 100 simulations with parameters $L = N\mu = 6$ and $p = 0.01$. (A) Model A; (B) Model B.

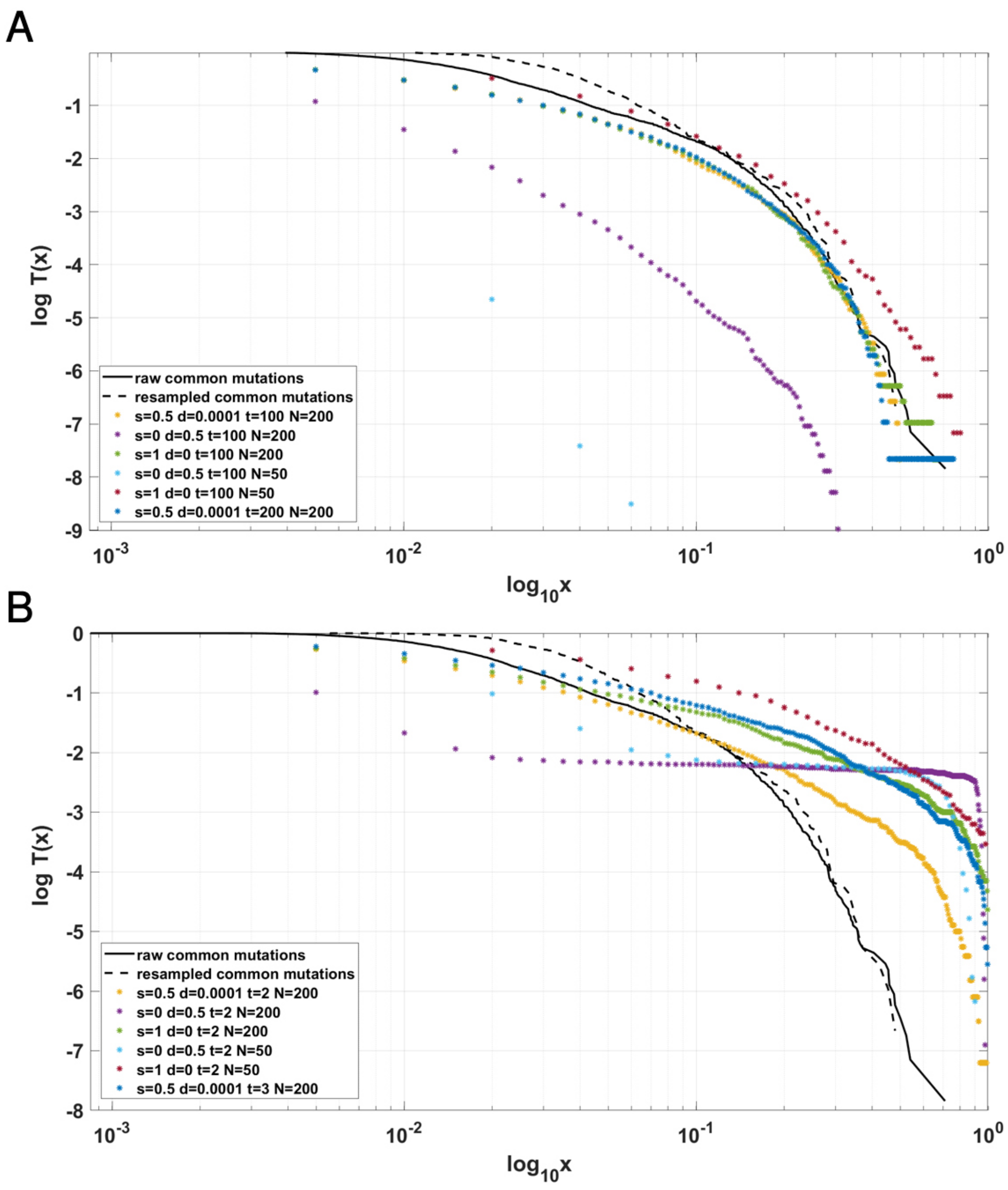

Figure S8. Comparison of log-log cumulative tails of the SFS obtained for experimental data from patient G31, to an average of 100 simulations with parameters $L = N\mu = 6$ and $p = 0.01$. (A) Model A; (B) Model B.

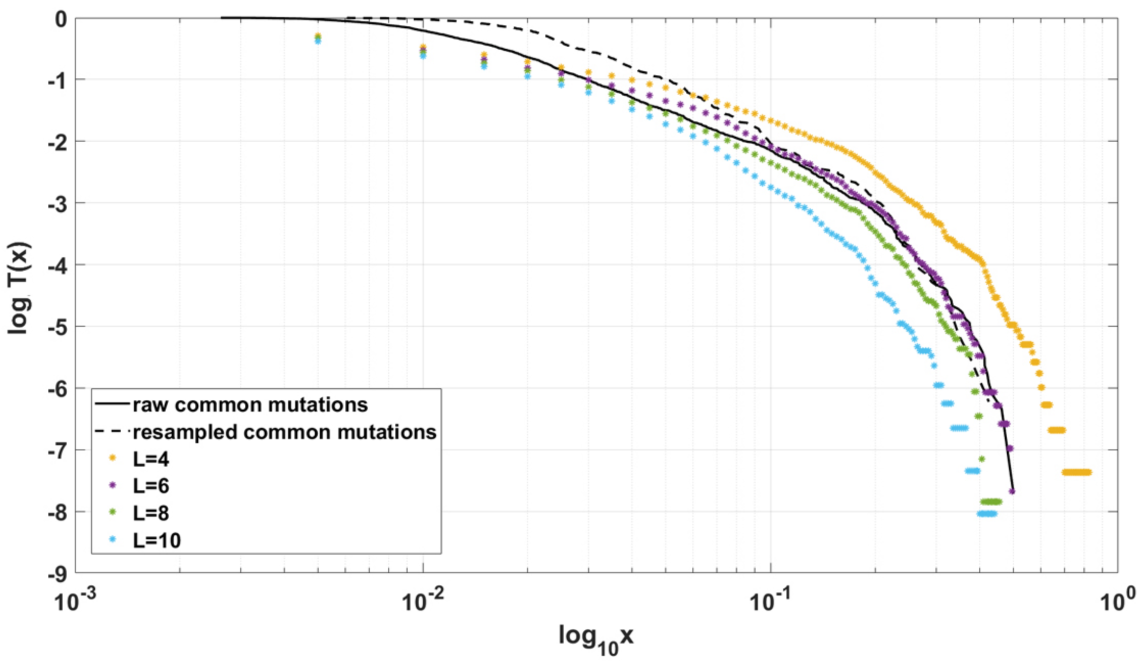

Figure S9. Comparison of log-log cumulative tails of the SFS obtained for experimental data from patient G30, to simulations using Model A. Average from 100 simulations on 200 cells at time $t = 100$. $s = 0.5$, $d = 0.0001$, $p = 0.01$, $N = 200$,

1.5 Comparison of semi-logarithmic cumulative tails of the SFS obtained for experimental data from patient G30 with simulations with Model A and Model B for a range of parameter sets.

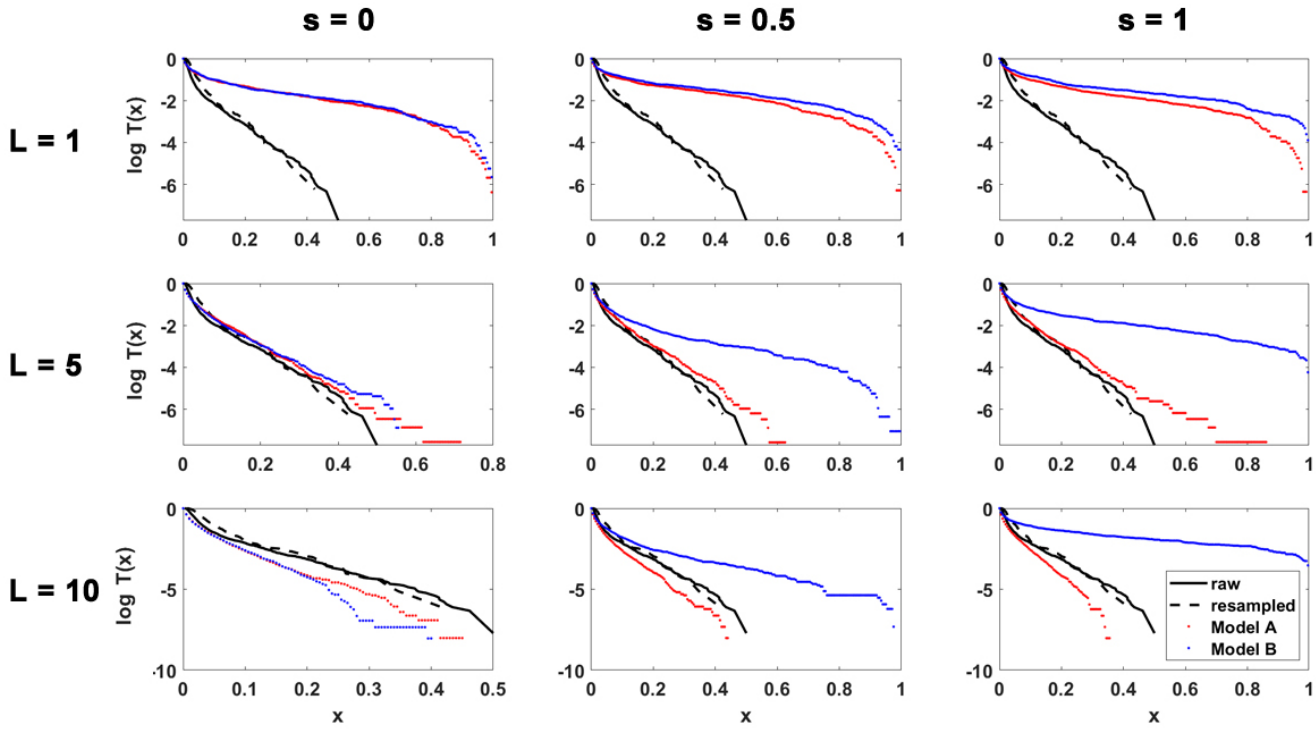

Figure S10. Comparison of semi-logarithmic cumulative tails of the SFS obtained for experimental data from patient G30 with simulations with Model A and Model B for a range of parameter sets. Average from 100 simulations on 200 cells at time $t = 100$. In all cases $d = 0$ and $p = 0.01$.

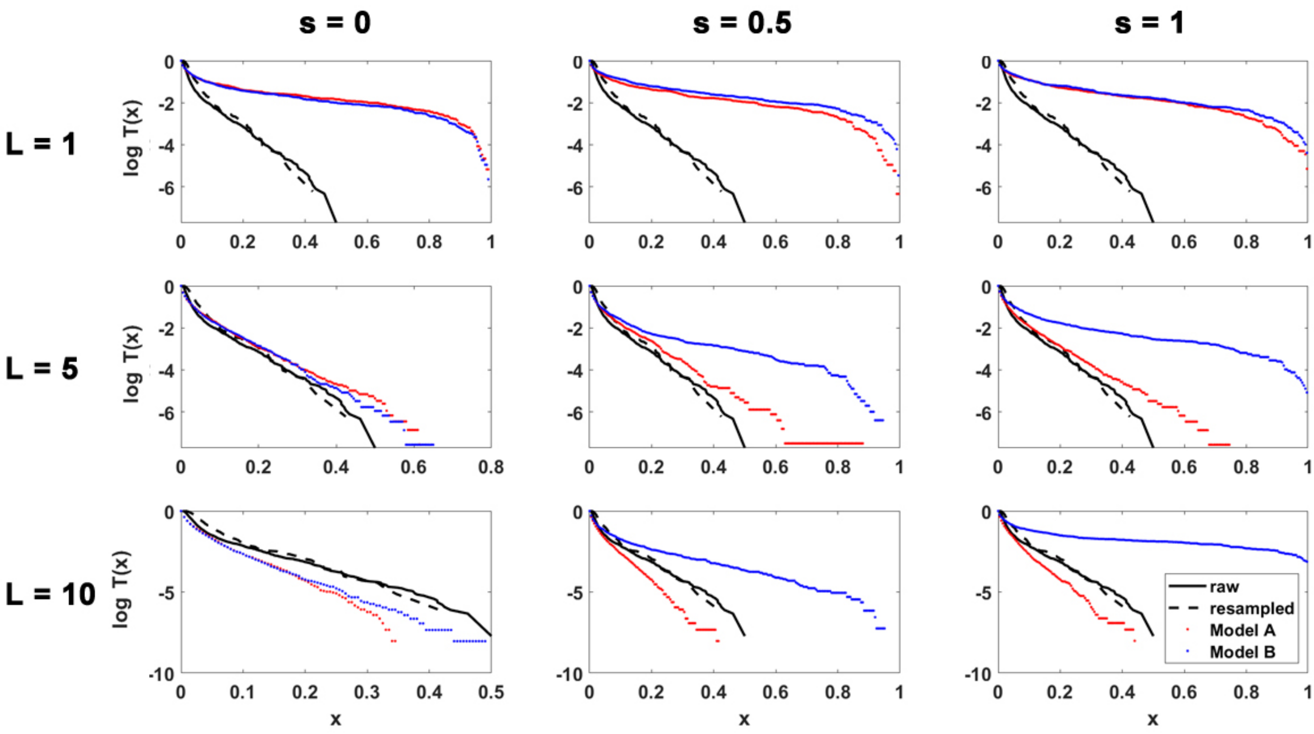

Figure S11. Comparison of semi-logarithmic cumulative tails of the SFS obtained for experimental data from patient G30, to simulations using Model A and Model B for a range of parameter sets. Average from 100 simulations on 200 cells at time $t = 100$. In all cases $d = 0.001$ and $p = 0.01$.

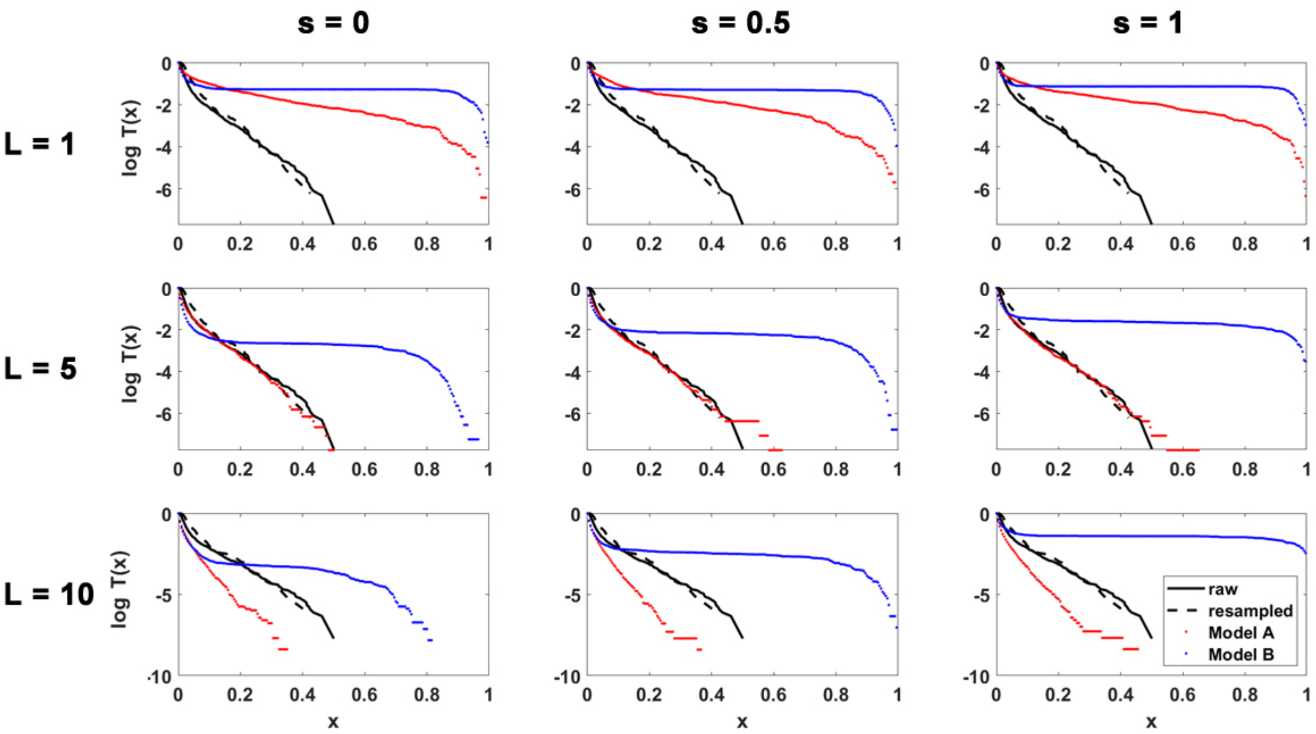

Figure S12. Comparison of semi-logarithmic cumulative tails of the SFS obtained for experimental data from patient G30, to simulations using Model A and Model B for a range of parameter sets. Average from 100 simulations on 200 cells at time $t = 100$. In all cases $d = 0.1$ and $p = 0.01$.

2 SUPPLEMENTAL INFORMATION: SITE FREQUENCY SPECTRA – COMPARISON OF VAF OF TCGA CANCER SPECIMENS TO MATHEMATICAL MODELS

In this section of Supplement, we compare the variant allele frequencies of selected TCGA tumors of breast, prostate, skin (melanoma), and ovary, to different models considered in the current paper. Breast and prostate cancers VAF histograms are predominantly ***unimodal***, while among cancers of lung and ovary, ***multimodal*** VAF histograms are quite common, and we focus on these for these two cancers.

For these reasons, we compare breast and prostate cancer VAFs to Models A and B, since the model of McDonald et al. (2018), as mathematically analyzed in Tung and Durrett (2021), seems to give SFS similar to Model B (Figure 13B in the main text). On the other hand, we compare the melanoma and ovarian cancers SFS to those generated by the Model of Dinh et al. (2020). Comparisons we carry out here do not have the ambition of being exhaustive. However, they seem to illustrate the diversity of possible models of evolution to be expected in different cancers.

We selected 2 specimens of each cancer type, and we focus on comparisons of the VAF cumulative tails $T(x)$ in the semi-logarithmic coordinates to their mathematically modelled SFS counterparts. As specified in the main body, comparison of cumulative tails, as opposed to probability mass functions or probability distribution functions, seems meaningful for several reasons; (i) the cumulative tails are smoother, (ii) they are all inscribed into a unit square, if VAF frequency $x = m/n$ is used as an argument, (iii) semi-logarithmic coordinates allow resolving differences in the “deep tail” ($T(x)$ small), and (iv) they are also less sensitive to differences in $T(x)$ for x small, which might be caused by DNA sequencing errors and data ”massaging”.

However, in the case of melanoma and ovarian cancer, we also supplement the model-fitted VAF histograms, to visualize how the model of Dinh et al. (2020) allows separating the clonal components.

As it is seen, we identified specimens of the breast and prostate cancers (Figures S13 and S14 respectively), which seem to have Model A or Model B - like SFS tails. We also found many breast and prostate cancers, which have SFS tails of types not fitting these models. Similarly, we identified multimodal SFS in melanoma and ovarian cancers (Figures S15 and S16 respectively), which are close to those generated by models in Dinh et al. (2020). Many of cancers of these two types have spectra not fitting models of Dinh et al. (2020). Please consult Discussion for further remarks.

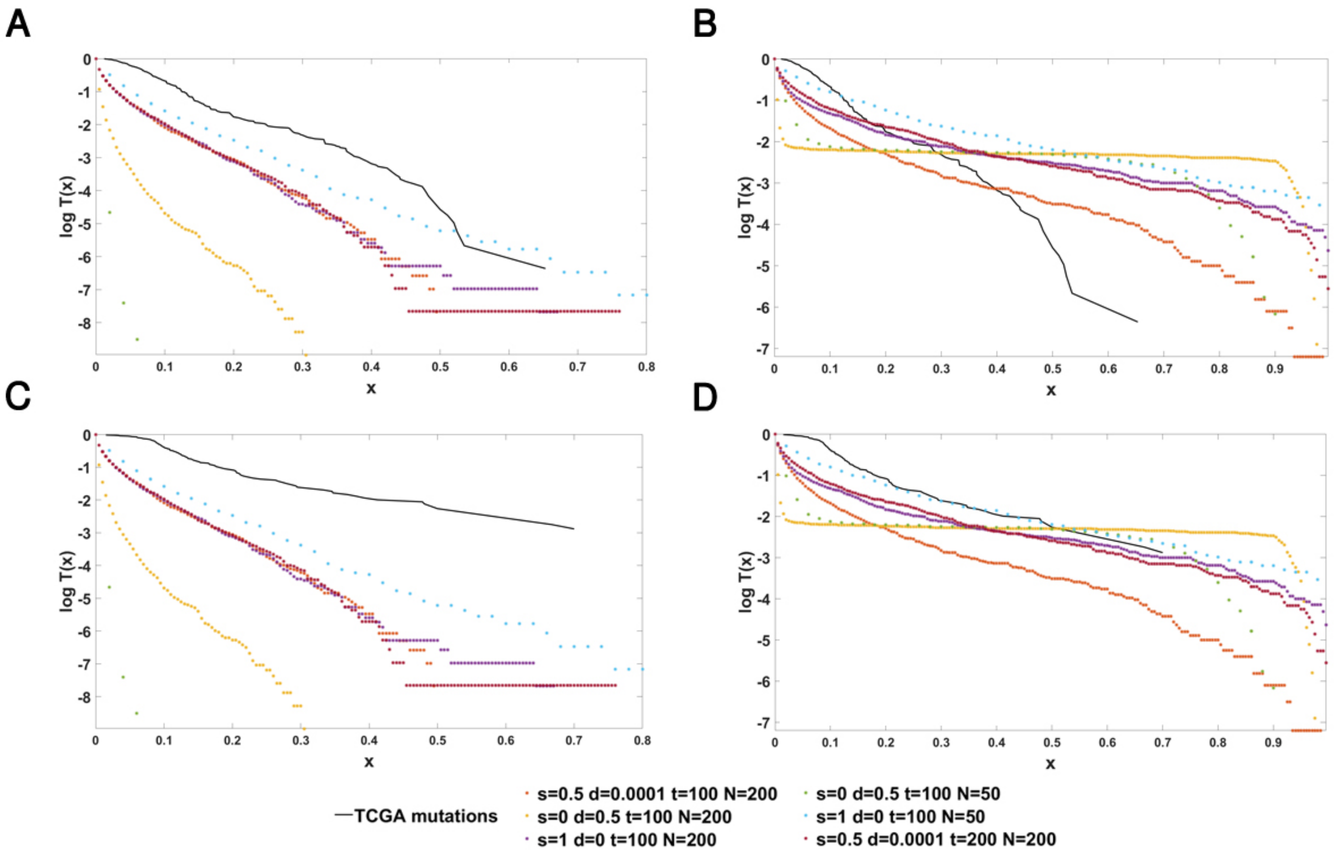

Figure S13. Comparison of the semi-logarithmic plots of the VAF cumulative tails of two breast cancers to their mathematically modelled counterparts. TCGA breast cancer TCGA-A8-A08H VAF, compared to $T(x)$ generated by Models A (A) and B (B). TCGA breast cancer TCGA-A0-A0JE VAF, compared to $T(x)$ generated by Models A (C) and B (D).

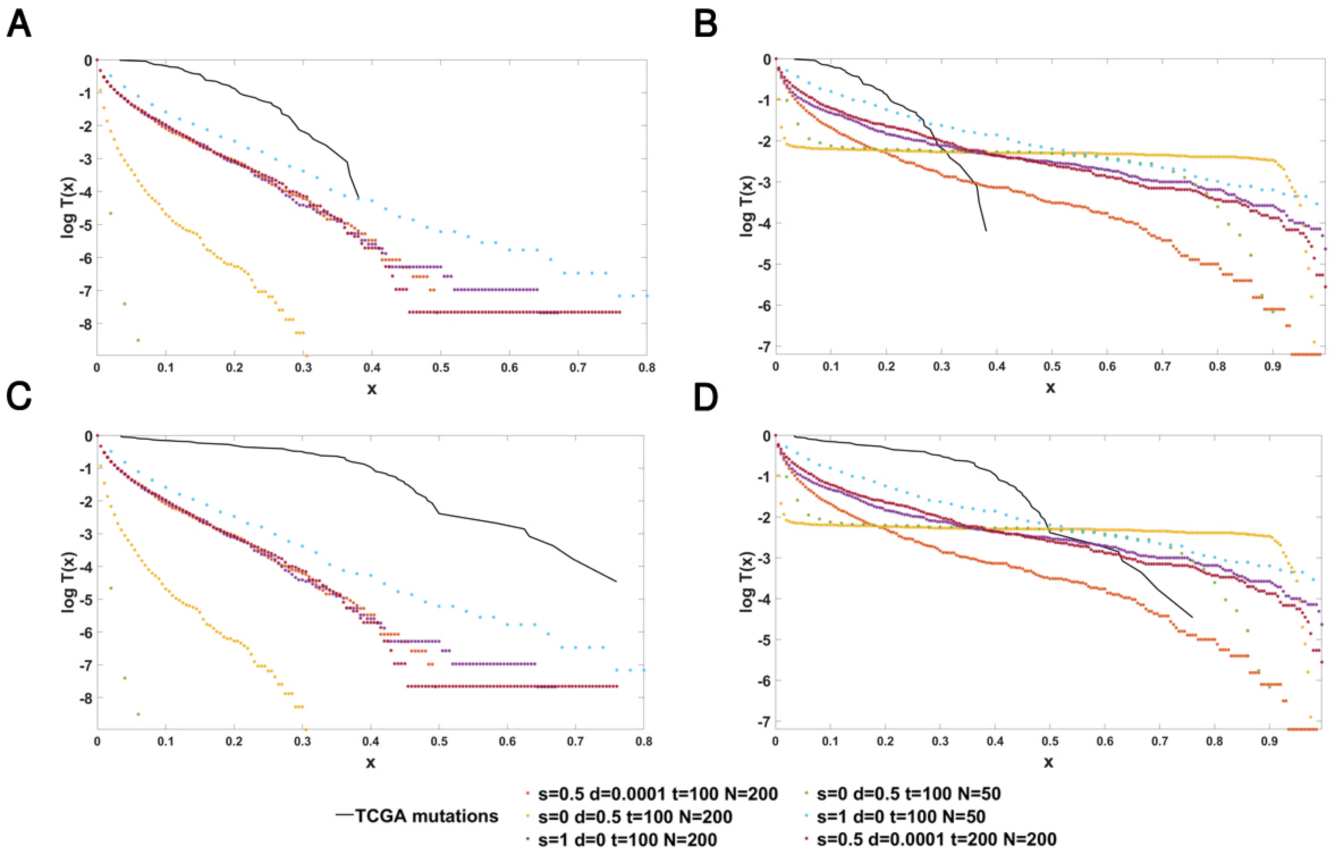

Figure S14. Comparison of the semi-logarithmic plots of the VAF cumulative tails of two prostate cancers to their mathematically modelled counterparts. TCGA prostate cancer TCGA-CH-5763 VAF, compared to $T(x)$ generated by Models A (A) and B (B). TCGA prostate cancer TCGA-EJ-5498 VAF, compared to $T(x)$ generated by Models A (C) and B (D).

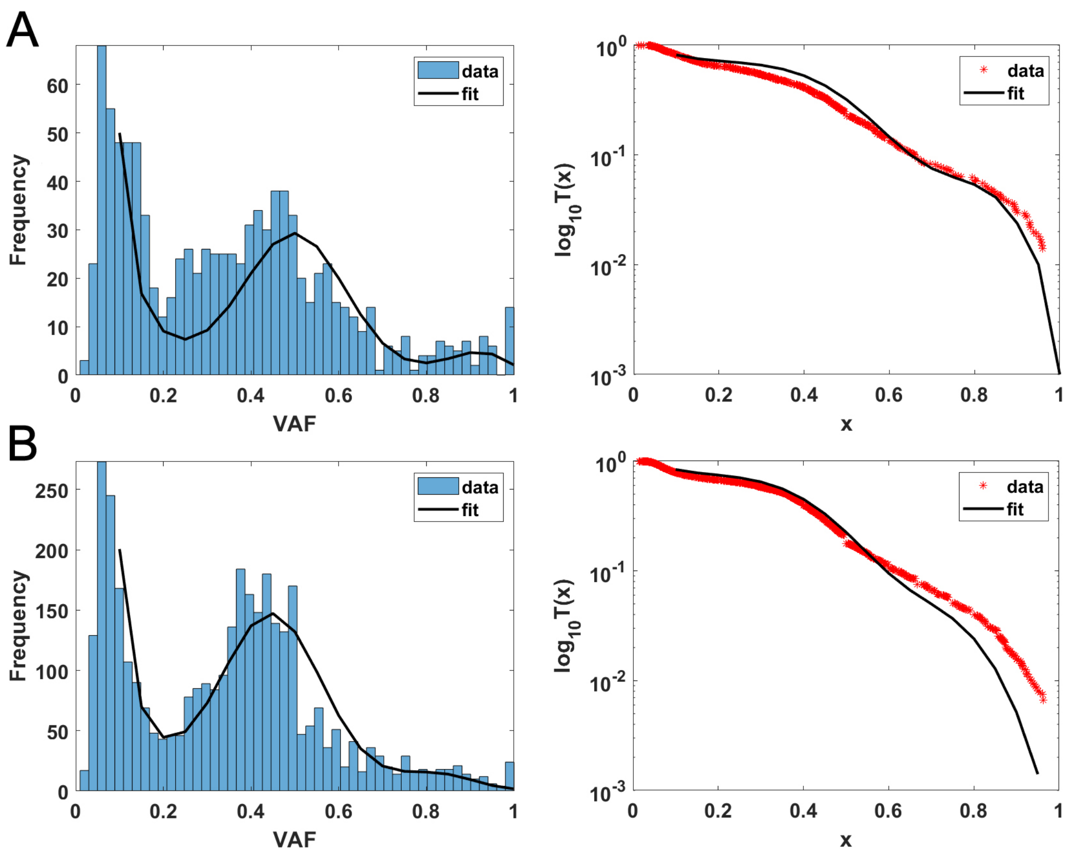

Figure S15. Comparison of the VAF histograms and semi-logarithmic VAF cumulative tails of two melanoma cancers to their mathematically modelled counterparts. (A) TCGA melanoma cancer TCGA-BF-AAOX VAF histograms and cumulative tails, compared to $S(x)$ and T(x) generated by Dinh et al. (2020) with parameters $A = 100$, $K1 = 15$, $K_2 = 160$, $p_0 = 0.1$, $p_1 = 0.4$, $p_2 = 0.5$, $P_1 = 0.9$, $P_2 = 0.5$. (B) TCGA melanoma cancer TCGA-D3-A2JG VAF histograms and cumulative tails, compared to $S(x)$ and $T(x)$ generated by Dinh et al. (2020) with parameters $A = 400$, $K_1 = 60$, $K_2 = 800$, $p_0 = 0.2$, $p_1 = 0.35$, $p_2 = 0.45$, $P_1 = 0.8$, $P_2 = 0.45$.

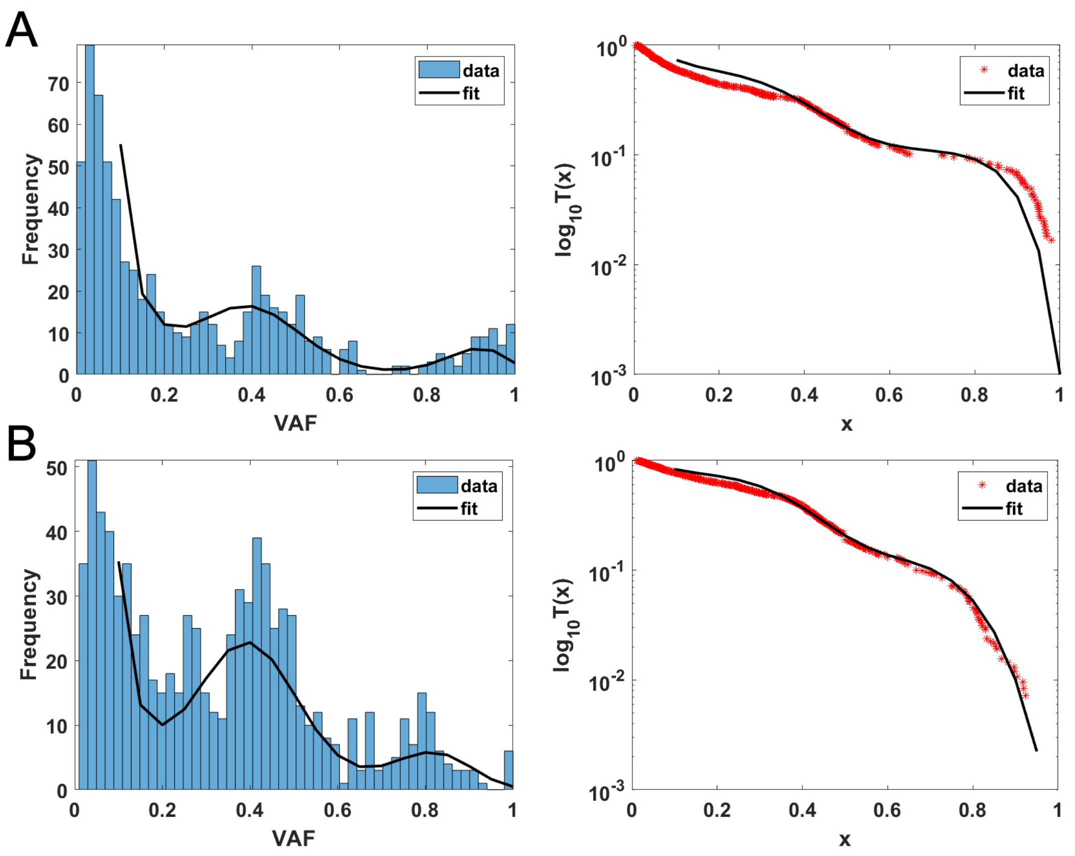

Figure S16. Comparison of the VAF histograms and semi-logarithmic VAF cumulative tails of two ovarian cancers to their mathematically modelled counterparts. (A) TCGA ovarian cancer TCGA-09-2050 VAF histograms and cumulative tails, compared to $S(x)$ and $T(x)$ generated by Dinh et al. (2020) with parameters $A = 110$, $K_1 = 20$, $K_2 = 80$, $p_0 = 0.1$, $p_1 = 0.4$, $p_2 = 0.5$, $P_1 = 0.9$, $P_2 = 0.4$. (B) TCGA ovarian cancer TCGA-13-1498 VAF histograms and cumulative tails, compared to $S(x)$ and $T(x)$ generated by Dinh et al. (2020) with parameters $A = 70$, $K_1 = 25$, $K_2 = 120$, $p_0 = 0.1$, $p_1 = 0.4$, $p_2 = 0.5$, $P_1 = 0.8$, $P_2 = 0.4$.

3 DNA SEQUENCING QUALITY CONTROL

The samples obtained from individuals G30 and G31 of the HER2+ breast cancer, which serve as an illustration for validity of Model A, were obtained from the collection of the Maria Sklodowska-Curie National Research Institute of Oncology, Krakow Branch, Poland, as a part of a larger study of the molecular evolution of breast cancer in Poland (as described in the main body of the paper). To obtain these, we commissioned a search of the archive of preserved specimens, all of which were paraffin-embedded (FFPE) samples. For this reason, direct validation using fast-frozen (FF) samples was impossible.

DNA was extracted from formalin-fixed paraffin-embedded (FFPE) tissues. DNA was isolated from tissue sections using a semi-automatic method available from Maxwell® RSC Instrument (Promega). The Maxwell® RSC DNA FFPE Kit (Promega) was used to extract genomic DNA as recommended by the manufacturer. The quality and amount of DNA was assessed using spectrophotometer NanoDrop 2000c and fluorimetrically using the Qubit™ dsDNA HS Assay Kit and Qubit 3.0 device (ThermoFisher Scientific).

Coverage charts (Figures S17 - S18) and a sheet with detailed statistics for these two samples (DNA sequencing quality control (table).xlsx) are provided. They list the read statistics for G30 and G31 tumors, subdivided into primary tumor (P), cancerous lymph node (L) and benign lymph node (C), used as control for the detection of somatic mutations.

Based on our experience the quality of data obtained based on the FFPE material, from patients G30 and G31, is comparable to the quality of data obtained typically using FF material. We were able to obtain $>$100x true median coverage for the regions captured by the Agilent SureSelect Human All Exon v7 probes, as shown on Figure S17. This was achieved both due to high quality of the reads, moderate read duplication rate for the total number of reads sequenced ($>$100 M/sample) and high mappability rate, as shown on Figure S18.

While we are unable to compare the results based on FFPE and FF samples obtained from the same individuals it seems safe to assume that for isolated DNA of good quality the results can be comparable between FFPE and FF, as shown in Astolfi et al. (2015).

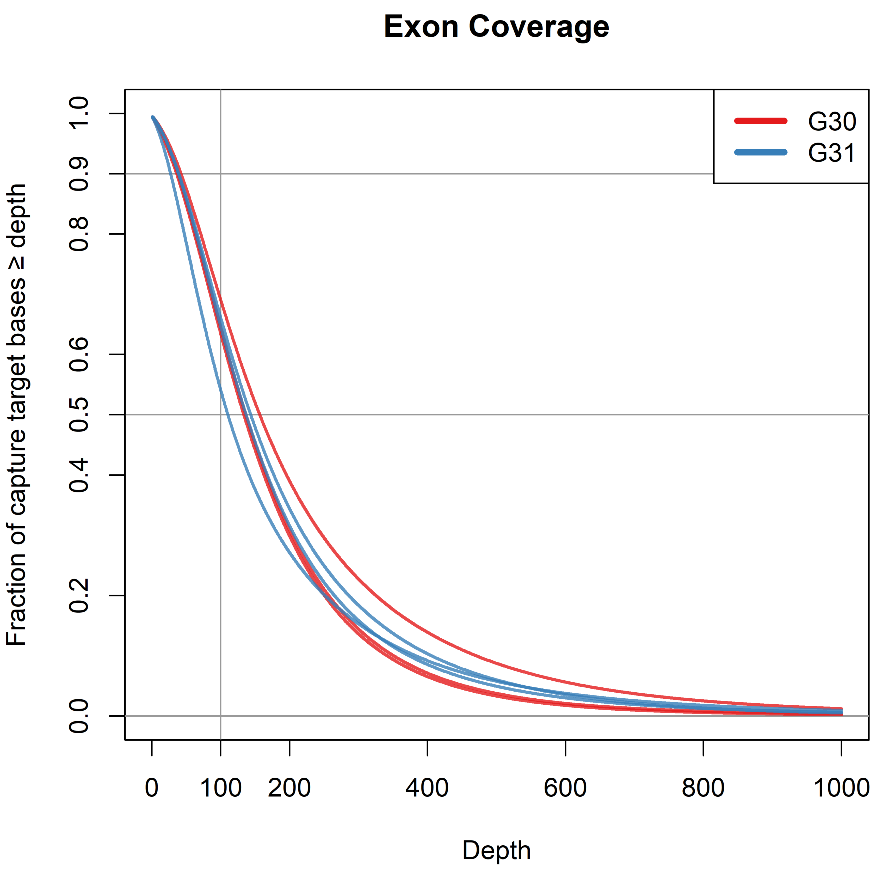

Figure S17. Exon coverage characteristics for the G30 (red) and G31 (blue) tumors.

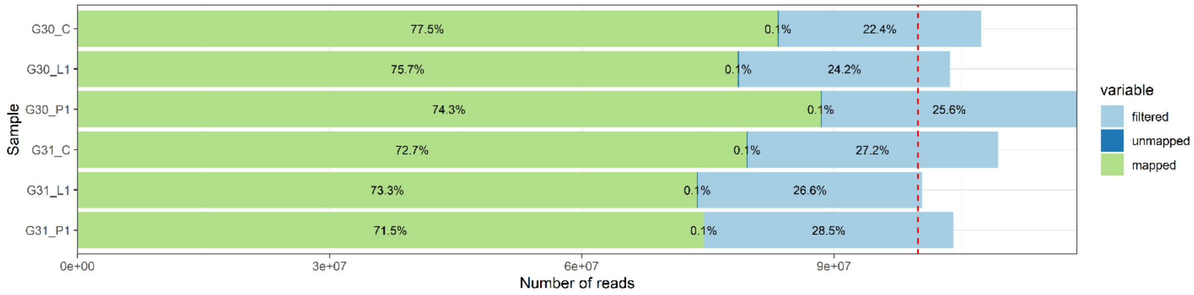

Figure S18. Reads statistics in the two G30 and G31 tumors.

REFERENCES

Astolfi, A., Urbini, M., Indio, V., Nannini, M., Genovese, C. G., Santini, D., et al. (2015). Whole exome sequencing (wes) on formalin-fixed, paraffin-embedded (ffpe) tumor tissue in gastrointestinal stromal tumors (gist). *BMC genomics* 16, 1–11

Dinh, K. N., Jaksik, R., Kimmel, M., Lambert, A., Tavaré, S., et al. (2020). Statistical inference for the evolutionary history of cancer genomes. *Statistical Science* 35, 129–144

McDonald, T. O., Chakrabarti, S., and Michor, F. (2018). Currently available bulk sequencing data do not necessarily support a model of neutral tumor evolution. *Nature genetics* 50, 1620–1623

Tung, H.-R. and Durrett, R. (2021). Signatures of neutral evolution in exponentially growing tumors: A theoretical perspective. *PLOS Computational Biology* 17, e1008701
